# Supplementary material for: Homogenizing interfacial assembly via indole-mediated binary monolayers for perovskite solar cells
Source: Nat Commun. 2026 Apr 2;17:4742. doi: 10.1038/s41467-026-71301-6 (PMC13216523; doi:10.1038/s41467-026-71301-6)
Supplement: Supplementary file 1 — Supplementary Information [file 41467_2026_71301_MOESM1_ESM.pdf]

Homogenizing Interfacial Assembly via Indole-Mediated Binary Monolayers for Perovskite Solar Cells

Haojiang Shen[1, †], Yeming Jin[1, †], Fuqiang Li[1, †], Kangning Zhao[2, †], Nan Shen[1, *], Jie Yang[1], Ya Li[1], Zhengyuan Long[1], Yuxuan Sheng[1], Hongbing Li[3], Fei Guo[3], Zong-Xiang Xu[4], Yong Ding[5], Xingzhu Wang[1,6 *], Geping Qu[7, *], Shi Chen[1, *], Mohammad Khaja Nazeeruddin[8,9 *]

[1]State Key Laboratory of Green Chemical Synthesis and Conversion, School of Energy Science and Technology, Henan Key Laboratory of Quantum Materials and Quantum Energy, School of Future Technology, Henan University, Zhengzhou, 450046, China

[2]School of Physical Science, Great Bay University, Dongguan, 523000, China

[3]Institute of New Energy Technology, College of Physics and Optoelectronic Engineering, Jinan University, Guangzhou, 510632, China

[4]Department of Chemistry, Southern University of Science and Technology, Shenzhen, Guangdong, 518055 China

[5]School of Renewable Energy, Hohai University, Nanjing, 211100 China

[6]Engineering and Research Center for Integrated New Energy Photovoltaics & Energy Storage Systems of Hunan Province, School of Electrical Engineering, University of South China, Hengyang, 421001, China

[7]Department of Materials Science and Engineering, City University of Hong Kong, Kowloon, Hong Kong, 999077, China

[8]Institut des Sciences et Ingénierie Chimiques, Ecole Polytechnique Fédérale de Lausanne (EPFL), CH-1015 Lausanne, Switzerland

[9]School of Integrated Circuits, Southeast University, Wuxi, Jiangsu, 214026, China

*Corresponding authors. Email: shennan@henu.edu.cn (N. S.); xingwang0926@163.com (X. W.); gepingqu@cityu.edu.hk (G. Q.);

chenshi@henu.edu.cn (S. C.); mdkhaja.nazeeruddin@epfl.ch (M. K. N.)
†These authors contributed equally to this work.

Supplementary Notes 1-10

Supplementary Figures 1-55

Supplementary Tables 1-9

Supplementary References 1-10

Supplementary Notes

Note 1 Calculation of interaction parameter (χ)[1]

Water and ethylene glycol (EG) are employed to determine the interaction parameters between small molecules (two-liquid method).

The surface tension (γ) is estimated by the Harmonic mean equations:

$$\gamma_{\mathrm{water}}(1+\cos\theta_{\mathrm{water}}) = 2(\gamma_{\mathrm{water}}^{\mathrm{d}})^{\frac{1}{2}} + 22(\gamma_{\mathrm{water}}^{\mathrm{p}}\gamma^{\mathrm{p}})^{\frac{1}{2}}$$

$$\gamma_{\mathrm{EG}}(1+\cos\theta_{\mathrm{EG}}) = 2(\gamma_{\mathrm{EG}}^{\mathrm{d}}\,\gamma^{\mathrm{d}})^{\frac{1}{2}} + 22\,(\gamma_{\mathrm{EG}}^{\mathrm{p}}\,\gamma^{\mathrm{p}})^{\frac{1}{2}}$$

$$\gamma = \gamma^{\mathrm{d}} + \gamma^{\mathrm{p}}$$

The interfacial tension between two different materials (e.g. A and B) can be calculated by the following equation:

$$\chi_{\mathrm{AB}} = \gamma_{\mathrm{A}} + \gamma_{\mathrm{B}} - 4\left(\frac{\gamma_{\mathrm{A}}^{\mathrm{d}}\gamma_{\mathrm{B}}^{\mathrm{d}}}{\gamma_{\mathrm{A}}^{\mathrm{d}}+\gamma_{\mathrm{B}}^{\mathrm{d}}} + \frac{\gamma_{\mathrm{A}}^{\mathrm{p}}\gamma_{\mathrm{B}}^{\mathrm{p}}}{\gamma_{\mathrm{A}}^{\mathrm{p}}+\gamma_{\mathrm{B}}^{\mathrm{p}}}\right)$$

where θ is the droplet contact angle on the neat film; γ_{AB} represents the interface tension between A and B; γ_{A} and γ_{B} are the surface tensions of pure films, respectively. The $\gamma_{\mathrm{A}}^{\mathrm{d}}$ and $\gamma_{\mathrm{A}}^{\mathrm{p}}$ are the dispersion and polar forces of A; The $\gamma_{\mathrm{B}}^{\mathrm{d}}$ and $\gamma_{\mathrm{B}}^{\mathrm{p}}$ are the dispersion and polar forces of B.

Note 2 Cylic voltammetry (CV) measurement[2,3]

Steps for CV evaluation: CV curves were obtained with a standard three-electrode system: a platinum net counter electrode, a silver reference electrode, and a glassy carbon working electrode. The SAM solutions were drop-casted onto the surface of the glassy carbon electrode and N_2-dried to form a film. The CV scans were then performed in an acetonitrile solution containing tetrabutylammonium hexafluorophosphate as the supporting electrolyte.

Highest occupied molecular orbital (HOMO) levels are calculated using empirical formula:

$$E_{\mathrm{HOMO}} = -e(E_{\mathrm{OX}} + 4.8 - E_{\mathrm{Fc/Fc^{+}}})$$

where E_{OX} represents the oxidation potential of the material; $E_{\mathrm{Fc/Fc^{+}}}$ is the formal potential of the Fc/Fc^{+} vs Ag/AgCl electrode (0.43 V in our study).

Steps for adsorption density evaluation: Glassy carbon working electrode was replaced by SAM-coated ITO and acetonitrile solution was replaced by 1,2-dichlorobenzene. The effective coverage of the self-assembled monolayers on the ITO surface is measured by the slope of the linear dependence of oxidation peak intensity and scan rate as follows:

$$i_{\mathrm{p,o}} = \frac{n^2F^2}{4RTN_{\mathrm{A}}}A\Gamma^*v$$

$i_{\mathrm{p,o}}$ is the oxidative peak current, v is the voltage scan rate, n is the number of electrons transferred, F is the Faraday constant (96,485 C mol^{-1}), R is the universal gas constant (8.314 J K^{-1} mol^{-1}), T is the temperature, N_A is the Avogadro constant, A is the electrode area, and Γ^* is the areal density.

Note 3 Micro stress analysis of thin films by the Williamson-Hall equation[4]

In Williamson-Hall analysis, the strain (ε) is derived from the following equation:

$$\beta\cos\theta = \frac{k\lambda}{D} + 4\varepsilon\sin\theta$$

where β is the crystallite size and can be calculated from the full width at half maximum (FWHM) of the peaks (100) (110) (200) (210) (211) (220), θ is Bragg diffraction angle, k is Scherrer constant, λ is X-ray wavelength, and D is the average thickness of the crystal grain.

Note 4 Carrier lifetime and differential lifetime calculation[5]

The carrier recombination lifetimes can be derived from the time-resolved photoluminescence (TRPL) by a bi-exponential fitting as shown in equation:

$$y = y_0 + A_1e^{\left(-\frac{\tau}{\tau_1}\right)} + A_2e^{\left(-\frac{\tau}{\tau_2}\right)}$$

where y_0 is constant, τ_1 and τ_2 are the decay components of the trap-assisted and radiative recombination process respectively. The average PL lifetime τ_{avg} can be calculated by equation:

$$\tau_{\mathrm{avg}} = \frac{A_1\tau_1^2 + A_2\tau_2^2}{A_1\tau_1 + A_2\tau_2}$$

We further use a differential lifetime (τ) to help distinguish the charge transfer more clearly as the following equation:

$$\tau = -\{d\ \ln[\varphi(t)]/\mathrm{d}t\}^{-1}$$

where φ(t) is the time-dependent PL photon flux. The differential lifetime was calculated by using fits to the TRPL transients.

Note 5 Calculation of surface recombination dynamics (SRV)[6]

The SRVs of our samples could be calculated with the following equation:

$$\tau_{\mathrm{avg}}{}^{-1} = \tau_{\mathrm{bulk}}{}^{-1} + \frac{SRV}{d}$$

where τ_{avg} is the average PL lifetime, τ_{bulk} is the PL lifetime obtained from perovskite on glass, and d (= 600 nm) is the perovskite film thickness.

Note 6 Calculation of quasi-Fermi level splitting (QFLS)[5]

QFLS can be calculated from PLQY by the following formula:

$$\mathrm{QFLS} = \mathrm{QFLS}_{\mathrm{rad}} + k_{\mathrm{B}}T\ \ln\left(\frac{J_{\mathrm{G}}}{J_{0,\mathrm{rad}}} \times \mathrm{PLQY}\right)$$

Where, $\mathrm{QFLS}_{\mathrm{rad}}$ is the radiation limit of semiconductor materials, k_B is the Boltzmann constant, T is the thermodynamic temperature. J_{G} is the photogenerated current density, $J_{0,\mathrm{rad}}$ is the dark state radiative recombination saturation current density. According to the detailed balance theory, the $J_{0,\mathrm{ra}d}$ can be calculated by the following equations:

$$J_{0,\mathrm{rad}} = q\int_0^{\infty} \mathrm{EQE}_{PV}(E)\ \Phi_{\mathrm{BB}}(E)\mathrm{d}E$$

$$\Phi_{\mathrm{BB}}(E) = \frac{2\pi E^2}{h^3c^2\ \exp\left(\frac{E}{K_{\mathrm{B}}T}\right) - 1}$$

Where q is the elementary charge, $\mathrm{EQE}_{\mathrm{PV}}$ is the photovoltaic external quantum efficiency, Φ_{BB} is the black-body radiative spectrum, E is the photo energy, h is the Planck constant, and c is the light speed in vacuum.

Note 7 Analyzation of space-charge-limited current (SCLC) model[7]

The dark *J-V* curves of the electron-only can be divided into three parts: Ohmic region, trap-filling limited region and the trap-free Child's region. The trap density (N_{t}) of perovskite films can be calculated from the equation:

$$N_{\mathrm{t}} = \frac{2\varepsilon\varepsilon_0 V_{\mathrm{TFL}}}{qL^2}$$

where q is the electron charge, d is the thickness of perovskite film, ε_0 and ε are the vacuum permittivity and relative dielectric constant of perovskite, and V_{TFL} is ordinate

of the intersection of ohmic region and trap-filling limited region. When the applied voltage is increased further, the current follows a linear relationship with the voltage squared (n=2), and application of the Mott-Gurney law allows us to obtain the carrier mobility (μ) using the following formula:

$$\mu = \frac{8J_{\mathrm{D}}L^3}{9\varepsilon\varepsilon_0V^2}$$

where J_{D} represents the current density and V represents the applied voltage, and the relative dielectric constants, vacuum dielectric constants, and thicknesses of the various SAM films are represented by ε, ε_0 and L, respectively.

Note 8 Trap density of states (t-DOS)[8]

The significant profile of trap density of states (tDOS) is deduced from the angular frequency dependent capacitance by the equation:

$$\mathrm{DOS}(E_\omega) = -\frac{V_{\mathrm{bi}}}{qW}\frac{dC}{dW}\frac{\omega}{k_BT}$$

$$\omega = 2\pi f$$

where C is the capacitance, ω is the angular frequency, f is the tuned frequency, q is the elementary charge, k_{B} is the Boltzmann constant and T is the temperature. V_{bi} and W are the built-in potential and depletion width, respectively, which were extracted from the Mott–Schottky analysis.

The capacitance-voltage (C-V) curve of PSCs in the Mott-Schottky configuration, and the calculation formula is as follows:

$$\frac{1}{C^2} = \frac{2(V_{\mathrm{bi}} - V)}{A^2q\varepsilon_0\varepsilon N_{\mathrm{A}}}$$

Where V_{bi} is the built-in potential, V is the applied voltage, A is the device active area, N_{A} is the doping concentration, q is the elementary charge, ε_0 is the vacuum permittivity, ε is dielectric constant.

The depletion width W can be calculated at 0 bias voltage:

$$W = \sqrt{\frac{2\varepsilon_0\varepsilon V_{\mathrm{bi}}}{qN_{\mathrm{A}}}}$$

The applied angular frequency ω defines an energetic demarcation:

$$E_\omega = k_BT\ln(\frac{\omega_0}{\omega})$$

where ω_0 is the attempt-to-escape frequency that equals to $2\pi\nu_0/T^2$. The ν_0 is temperature-independent attempt-to-escape frequency according to the equation $\ln(T^2/\omega) = E_T/kT - \ln(2\pi\nu_0)$ obtained at different T. ω_0 the attempt-to-escape frequency of 3.4×10^{11} Hz for $Cs_{0.05}FA_{0.85}MA_{0.1}PbI_3$[9]. All the tests were conducted in dark conditions.

Note 9 The dependence of V_{OC} of the PSCs on light intensity[10]

The ideality factor nk_BT/q can be calculated by equation:

$$V_{\mathrm{OC}} = \frac{nk_{\mathrm{B}}T}{q} ln(P_{\mathrm{light}})$$

Note 10 Fill factor (FF) loss analysis[11]

The FF losses in high-performing PSCs are determined by two main factors, non-radiative loss and charge transport loss. The maximum FF (FF_{max}) can be empirically calculated according to the following equation:

$$FF_{\mathrm{max}} = \frac{V_{\mathrm{OC}} - ln(V_{\mathrm{OC}} + 0.72)}{V_{\mathrm{OC}} + 1}$$

Where $V_{\mathrm{OC}} = \frac{V_{\mathrm{OC}}}{nk_{\mathrm{B}}T/q}$ (n is ideality factor, k_B is Boltzmann constant, T is temperature, q is elementary charge).

Supplementary Figures

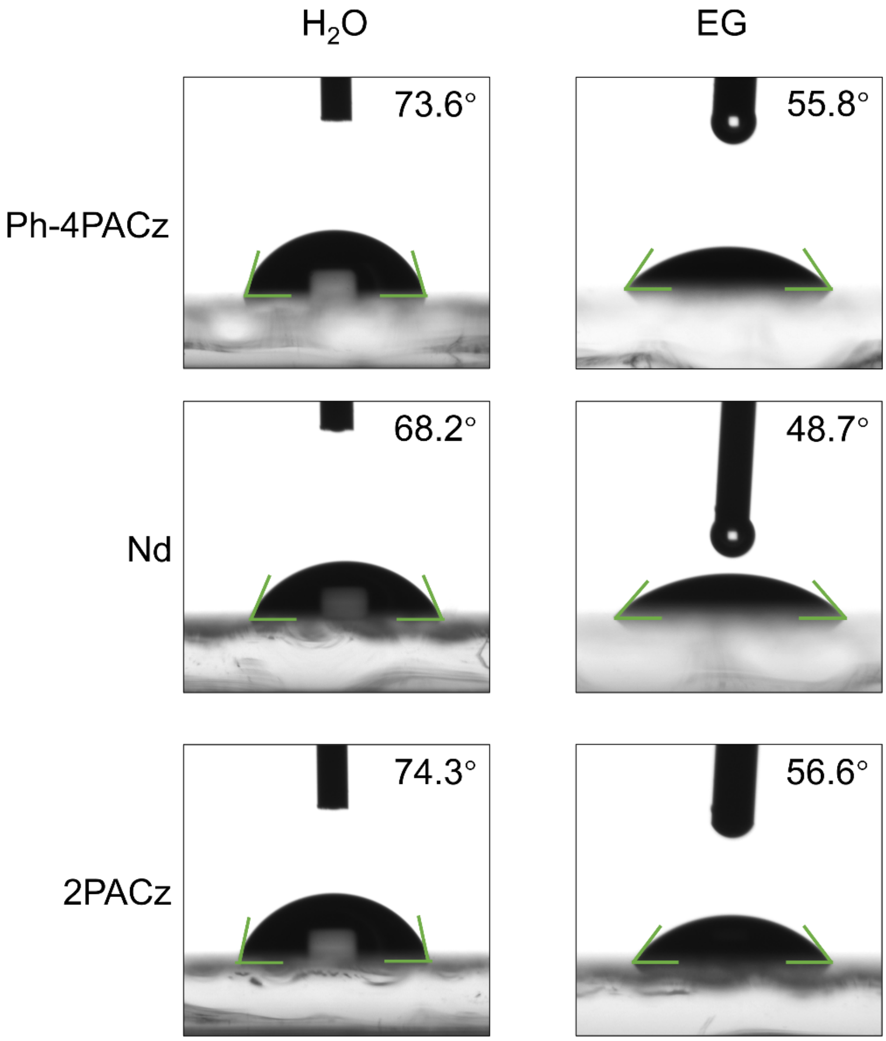

Supplementary Fig. 1 Contact angle measurements of Ph-4PACz, Nd and 2PACz for water and ethylene glycol (EG). Water and ethylene glycol were selected as testing solvents because they have well-separated polar surface tension and dispersive surface tension. This is essential for accurate surface energy deconvolution based on the Owens-Wendt-Rabel-Kaelble (OWRK) model (*Polym. Bull.* **25**, 265-271, (1991), *Joule* **4**, 1278-1295, (2020), *Nat. Energy* *7*, 1180-1190 (2022)), therefore delivering a precise interaction parameter. The interaction parameter between Ph-4PACz and 2PACz was determined to be 0.16, indicating their good miscibility. However, such good miscibility does not inhibit the inherent SAM self-aggregation due to their same functional groups (carbazole and phosphate).

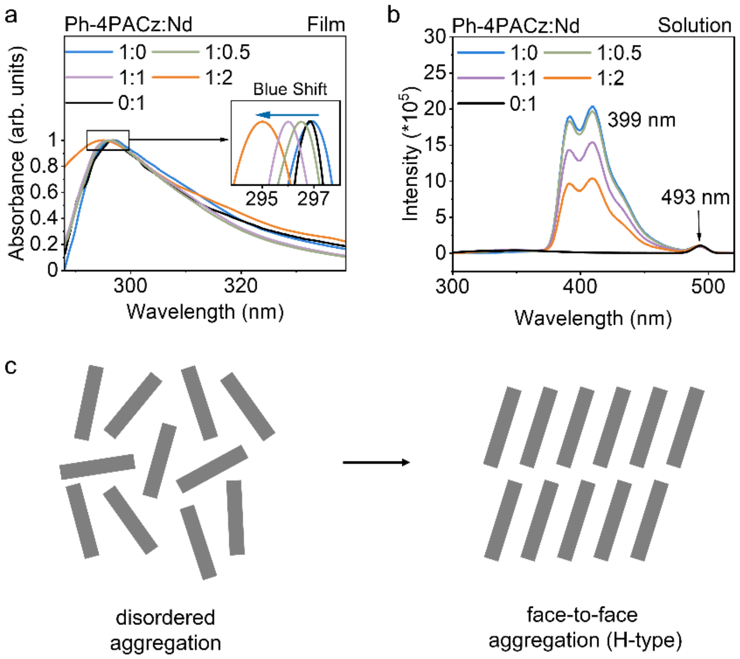

Supplementary Fig. 2 **a** UV-Vis spectra of Ph-4PACz films without and with Nd (different molar ratios). **b** PL spectra of Ph-4PACz, Nd and Ph-Nd in dichloromethane solution. **c** Schematic illustration of the transformation of the intermolecular aggregation behavior from a disordered aggregation in Ph-4PACz to an ordered face-to-face aggregation (H-type) in Ph-Nd. Ph-4PACz and Nd have the same concentration of 1×10^{-5} M while all the Ph-Nd solutions contain the same Ph-4PACz concentration of 1×10^{-5} M. Under 254 nm excitation, Ph-4PACz and Ph-Nd solution samples display a dominant emission peak at approximately 399 nm, while Nd solution sample lacks such emission under this excitation. All samples have the 493 nm signal identified as the second harmonic artifact.

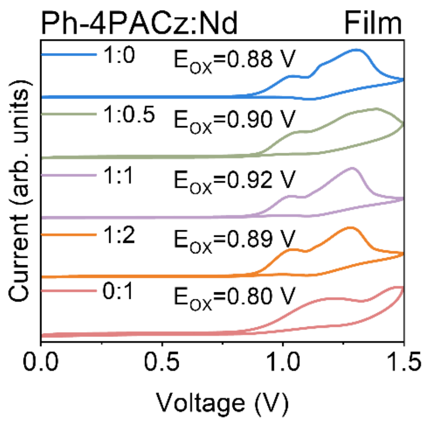

Supplementary Fig. 3 CV curves of Ph-4PACz without and with Nd (different molar ratios).

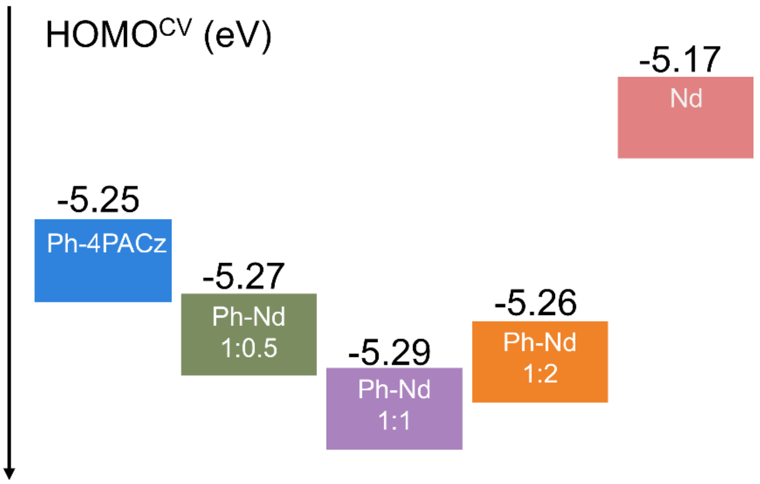

Supplementary Fig. 4 HOMO levels extracted from CV measurements.

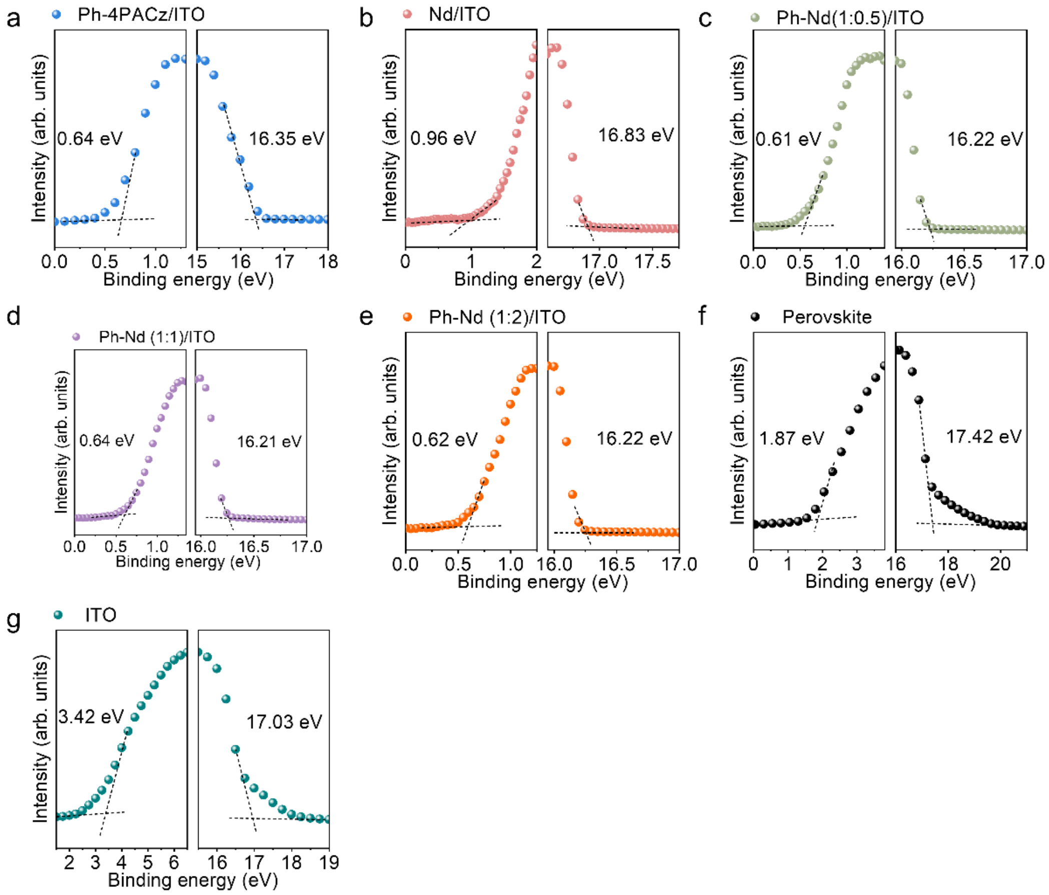

Supplementary Fig. 5 UPS spectra of **a** Ph-4PACz, **b** Nd, **c** Ph-Nd (1:0.5), **d** Ph-Nd (1:1), **e** Ph-Nd (1:2), **f** perovskite films deposited on ITO and **g** bare ITO.

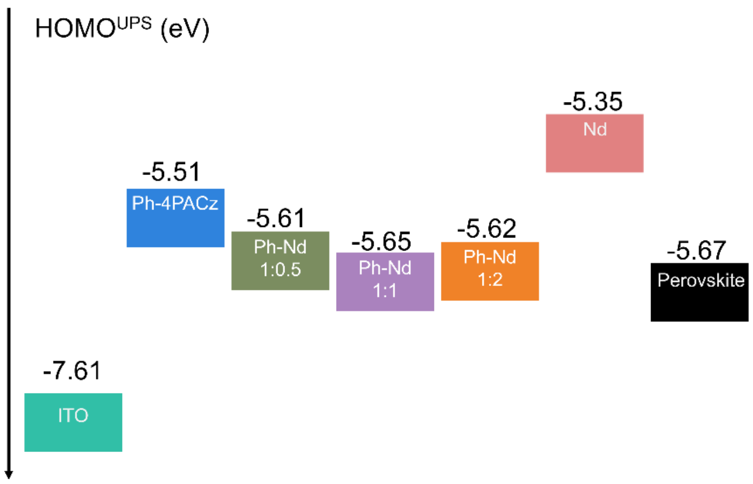

Supplementary Fig. 6 HOMO levels extracted from UPS measurements. The enhanced π-π interaction and hydrogen bonding in Ph-Nd lead to the redistribution of the electron cloud between Ph-4PACz and Nd, where the HOMO and LUMO are localized on the Ph-4PACz and Nd components, respectively (namely frontier orbital rearrangement). Consequently, the co-adsorbed SAMs exhibit a novel electronic structure featuring with nonlinear HOMO levels (−5.61, −5.65 and −5.62 eV for the 1:0.5, 1:1, and 1:2 Ph-4PACz/Nd), much deeper than those (−5.51 and −5.35 eV) of pure Ph-4PACz and Nd.

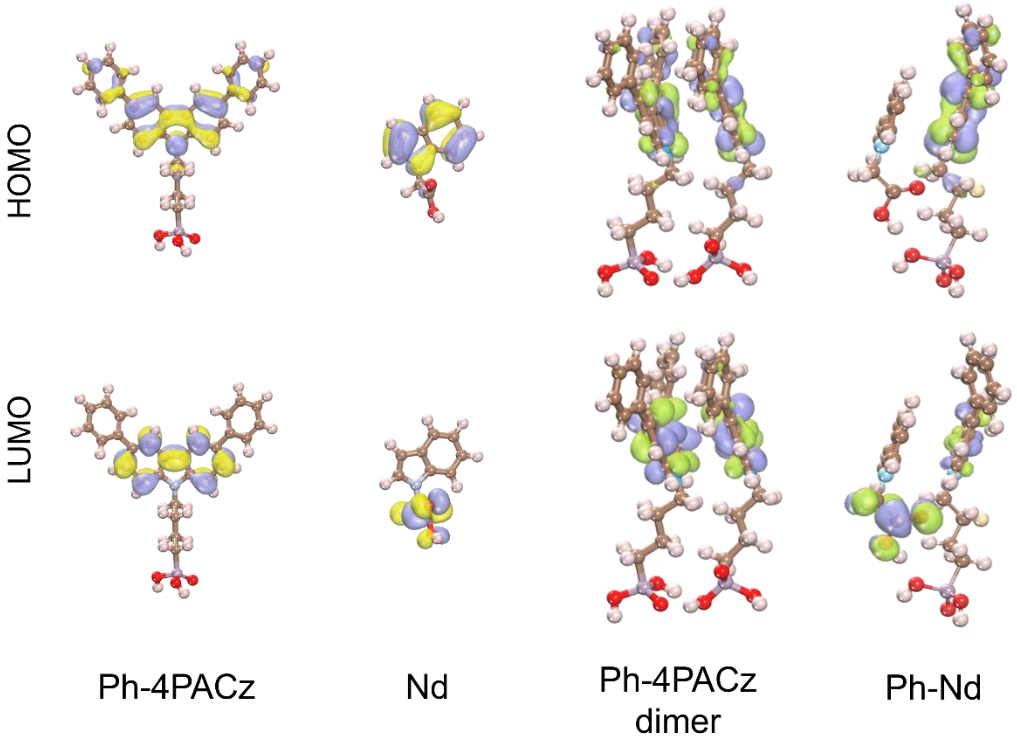

Supplementary Fig. 7 Molecular orbital distributions of Ph-4PACz, Nd, Ph-4PACz dimer and Ph-Nd dimer. The frontier orbitals of Ph-4PACz, Nd and Ph-4PACz dimer are localized within individual molecules. In contrast, for the Ph-Nd dimer, frontier orbital rearrangement occurs, with the HOMO and LUMO localized on Ph-4PACz and Nd parts, respectively.

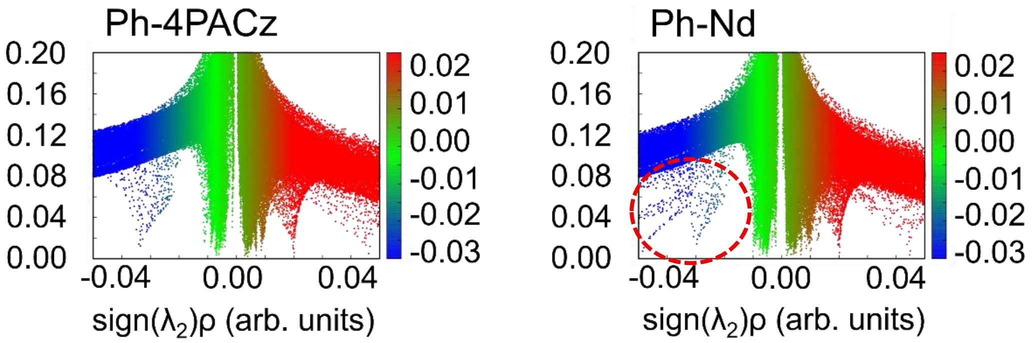

Supplementary Fig. 8 IGMH maps of **a** Ph-4PACz dimer and **b** Ph-Nd dimer. The blue region denotes areas of attractive forces, primarily arising from π-π interactions and hydrogen bonds[12].

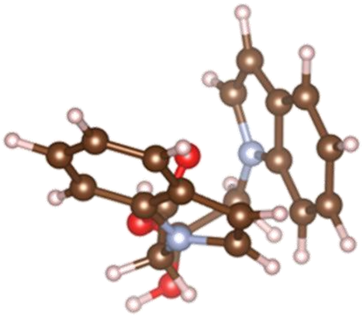

Nd

Supplementary Fig. 9 Optimized structure of the Nd dimer.

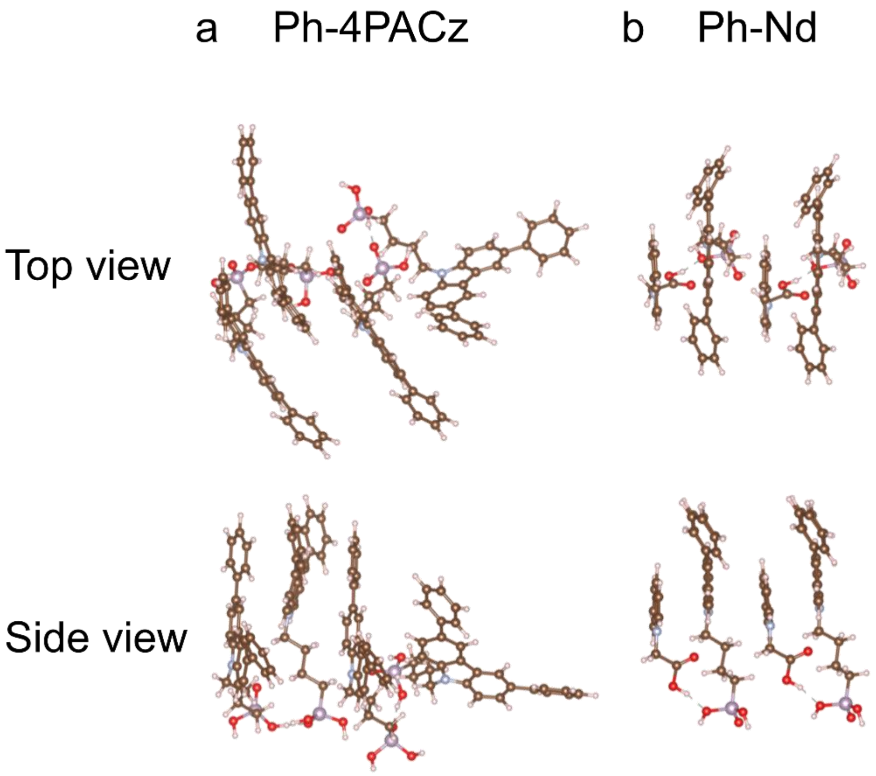

Supplementary Fig. 10 Optimized structures of **a** Ph-4PACz and **b** Ph-Nd tetramers.

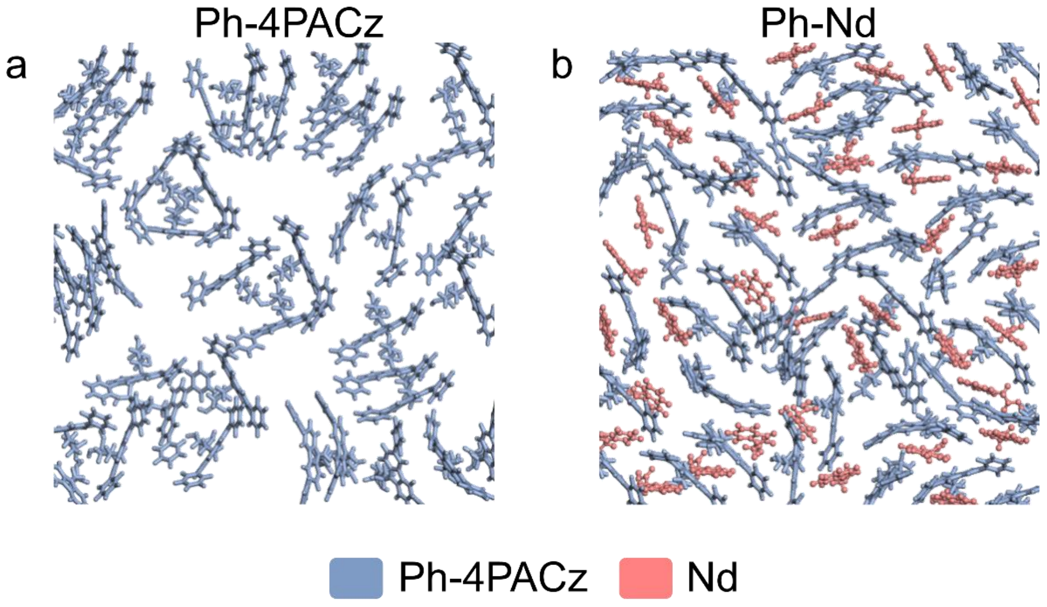

Supplementary Fig. 11 Optimized structures of **a** Ph-4PACz and **b** Ph-Nd clusters (70 Å×70 Å×30 Å).

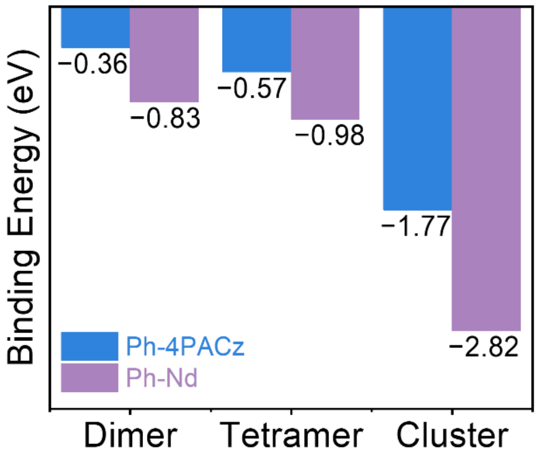

Supplementary Fig. 12 Binding energy of Ph-4PACz and Ph-Nd dimers, tetramers and clusters.

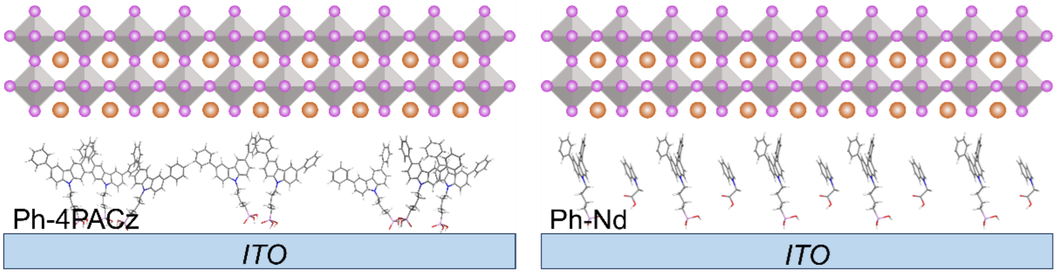

Supplementary Fig. 13 Schematic illustrations of the molecular arrangement in Ph-4PACz and Ph-Nd.

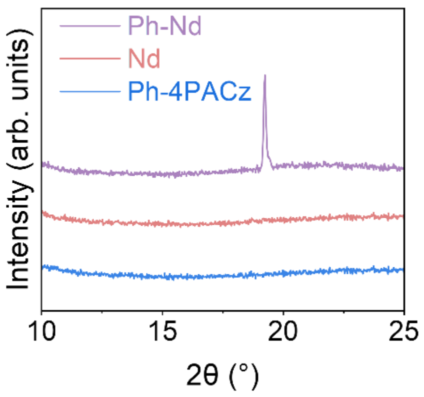

Supplementary Fig. 14 XRD patterns of Ph-4PACz, Nd and Ph-Nd films.

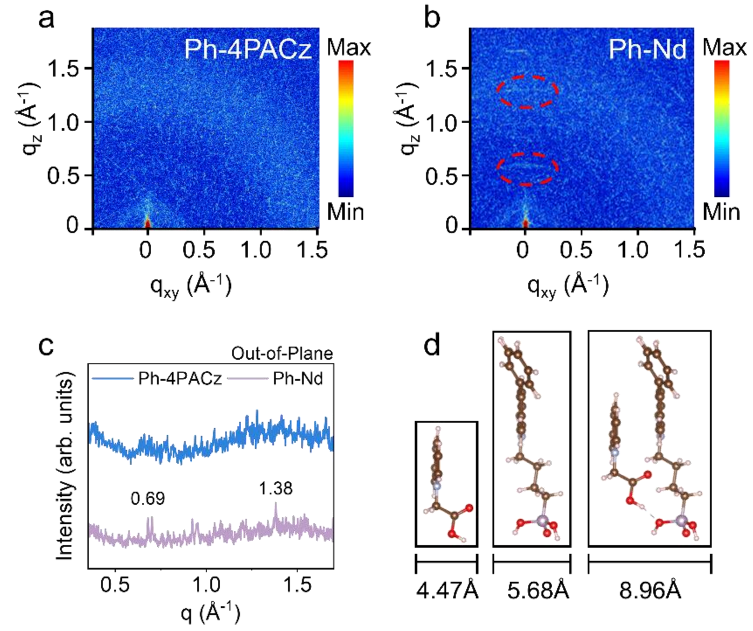

Supplementary Fig. 15 2D GIWAXS images of **a** Ph-4PACz and **b** Ph-Nd films. **c** 1D GIWAXS intensity profiles integrated from the 2D patterns. **d** Schematic illustration of the Ph-Nd dimer with the calculated parameters. The SAM samples for GIWAXS measurement were prepared on the ITO glass through the normal method.

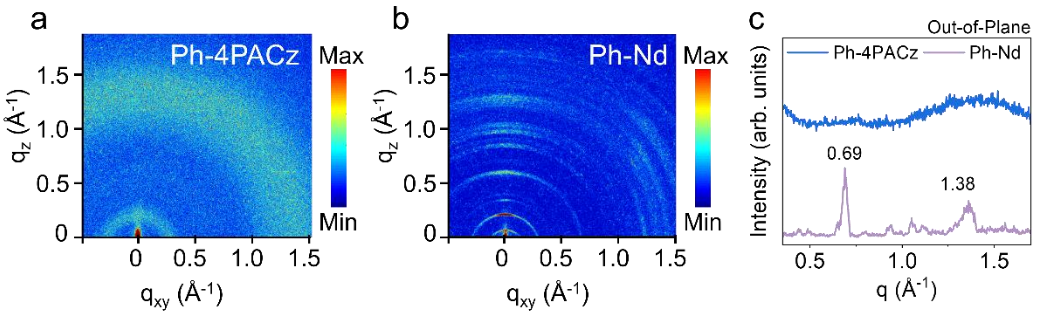

Supplementary Fig. 16 2D GIWAXS images of **a** Ph-4PACz and **b** Ph-Nd films (4 mg/ml samples). **c** 1D GIWAXS intensity profiles integrated from the 2D patterns. Such SAM samples for GIWAXS measurement were prepared on the ITO glass using the high-concentration SAM solutions through the normal method.

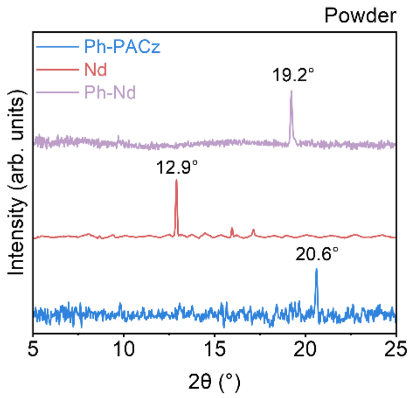

Supplementary Fig. 17 XRD patterns of Ph-4PACz, Nd and Ph-Nd powders. Ph-4PACz, Nd and Ph-Nd were recrystallized in n-hexane/tetrahydrofuran (4:1, 40 °C, overnight) solution system to obtain solid-state SAMs for XRD measurement.

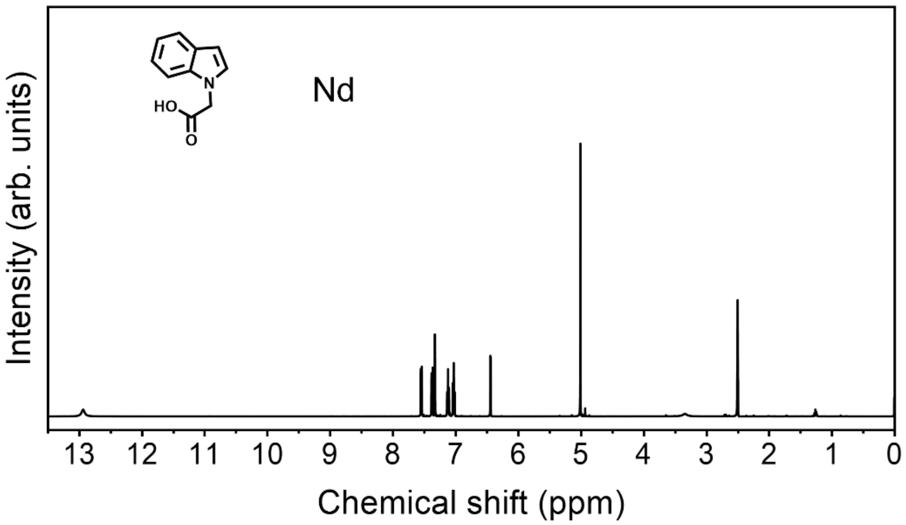

Supplementary Fig. 18 Liquid-state ^{1}H NMR spectra of Nd (d-DMSO).

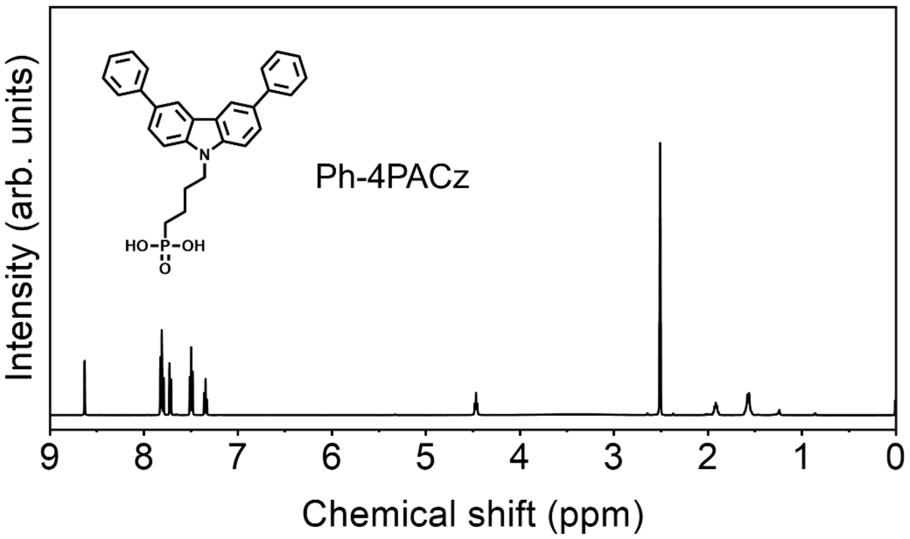

Supplementary Fig. 19 Liquid-state ^{1}H NMR spectra of Ph-4PACz (d-DMSO).

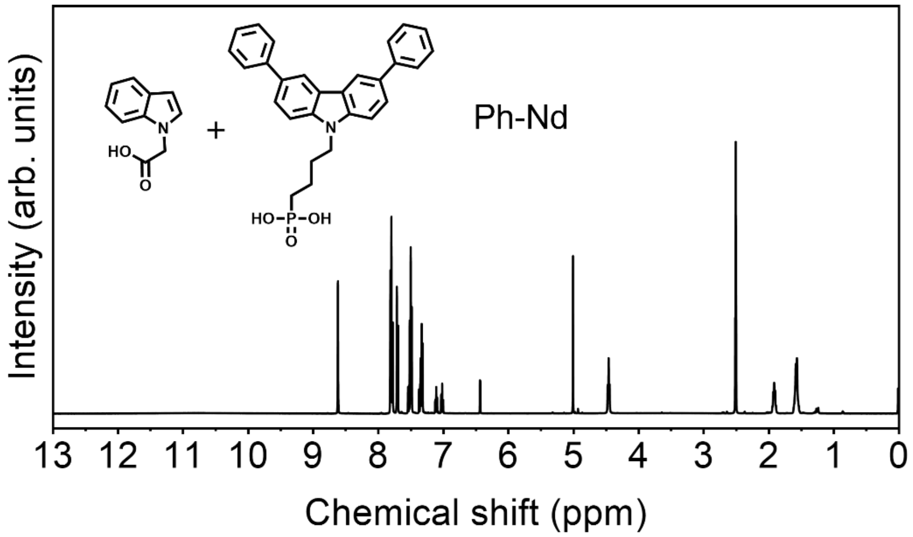

Supplementary Fig. 20 Liquid-state ^{1}H NMR spectra of Ph-Nd (d-DMSO).

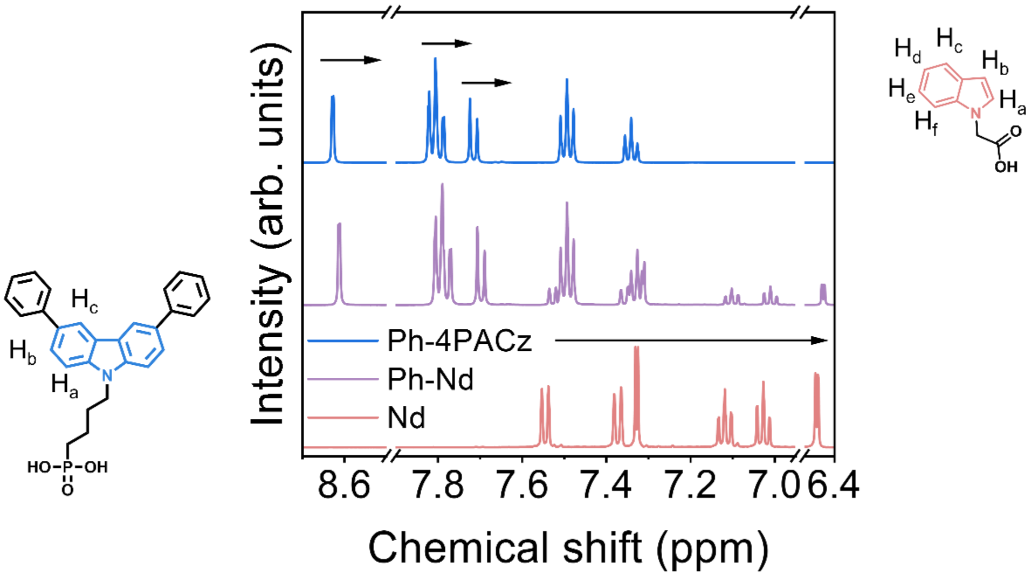

Supplementary Fig. 21 Partial enlarged ^{1}H NMR spectra of Ph-4PACz, Nd and Ph-Nd solutions. The characteristic peaks in the low-filed region (6-9 ppm) correspond to the protons on conjugated units (carbazole and indole rings).

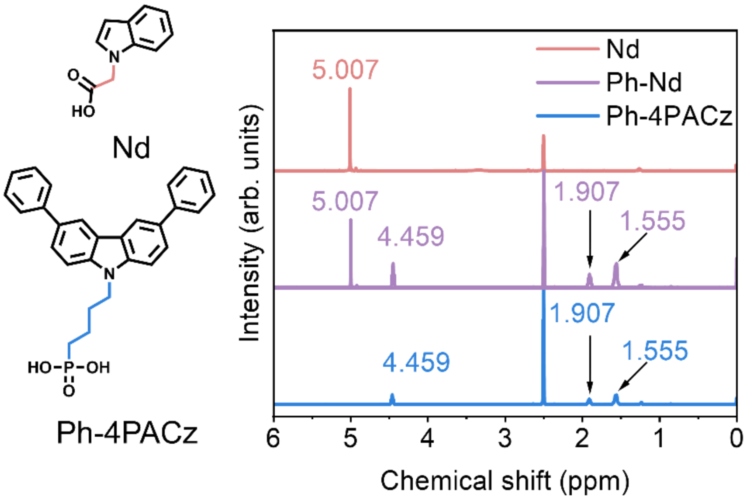

Supplementary Fig. 22 Partial enlarged ^{1}H NMR spectra of Ph-4PACz, Nd and Ph-Nd solutions. The characteristic peaks in high-filed region (< 6 ppm) correspond to the protons on alkyl parts (methylene groups).

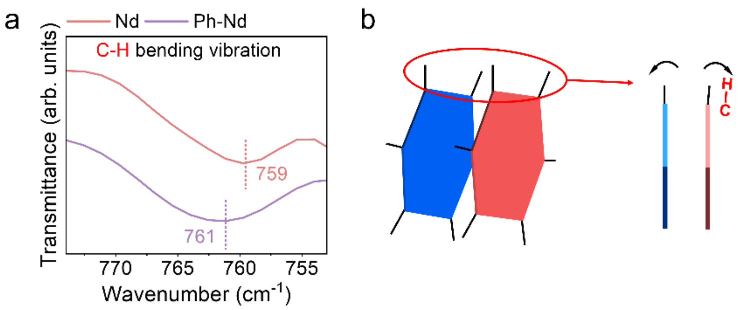

Supplementary Fig. 23 a Partial enlarged FTIR spectra of Nd and Ph-Nd powders. **b** Schematic of aromatic C-H bending vibration. Such shifts in the C-H bending peaks (including Fig. 2c) can be attributed to π-π interaction-induced perturbations of aromatic C-H bending modes in Ph-Nd, arising from steric constraints and electronic redistribution upon face-to-face stacking. This in turn confirms π-π interactions between the conjugated aromatic moieties of both Ph-4PACz and Nd.

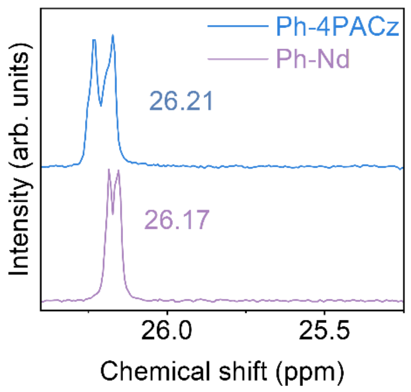

Supplementary Fig. 24 ^{31}P NMR spectra of Ph-4PACz and Ph-Nd solutions (d-DMSO).

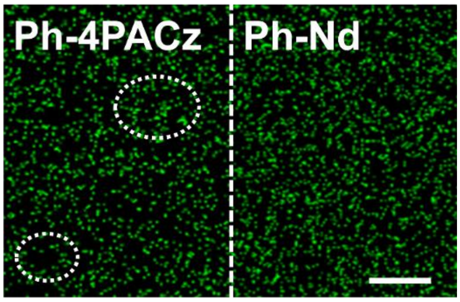

Supplementary Fig. 25 P mapping images of Ph-4PACz and Ph-Nd films on ITO (scale bar: 1 μm).

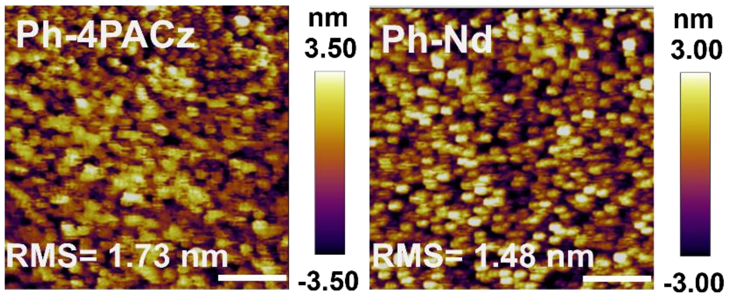

Supplementary Fig. 26 AFM images of Ph-4PACz and Ph-Nd films (scale bar: 1 μm).

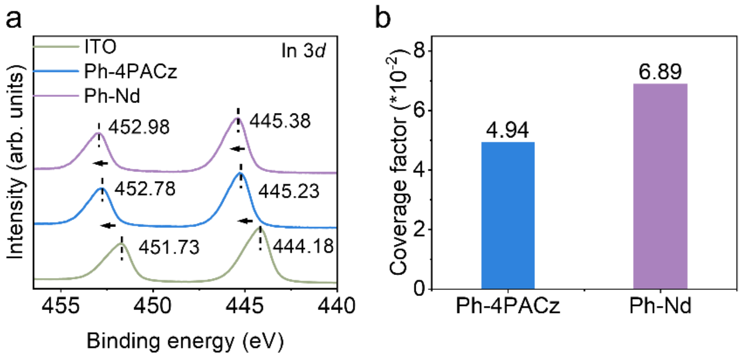

Supplementary Fig. 27 XPS spectra of In 3*d* for bare ITO, Ph-4PACz and Ph-Nd films. **b** Coverage factor values of Ph-4PACz and Ph-Nd films.

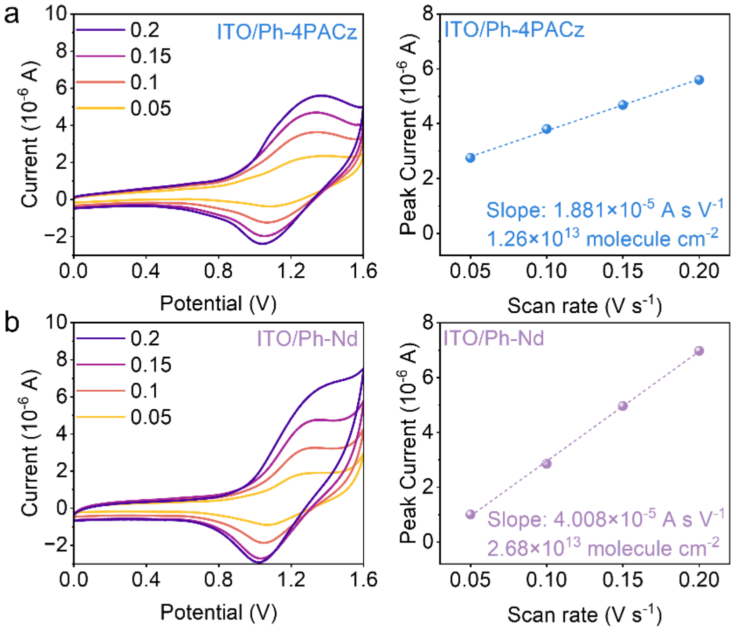

Supplementary Fig. 28 Cyclic voltammograms of **a** ITO/Ph-4PACz and **b** ITO/Ph-Nd as the working electrode measured in N_2-saturated o-DCB solution under different voltage scan rates and the corresponding relationship between the oxidative peak current and the voltage scan rate. The Ph-4PACz area density in Ph-Nd system is half of the total area density (1.34×10^{13} molecule cm^{-2}).

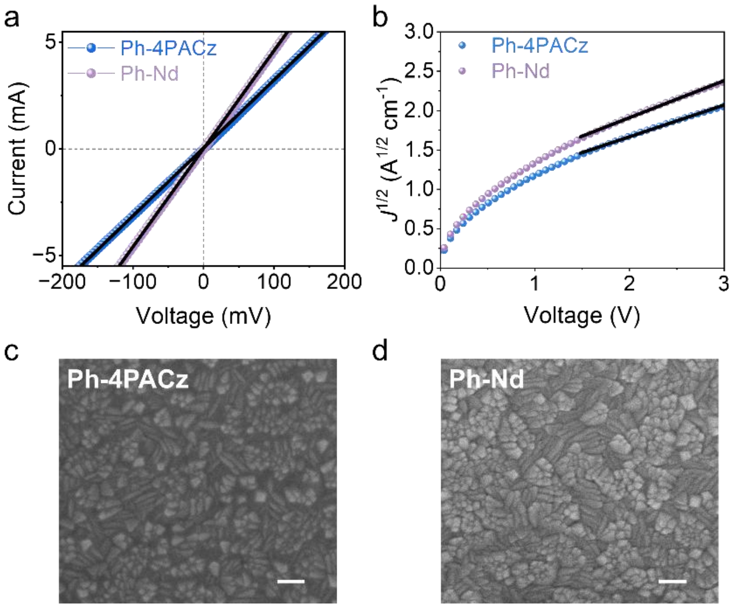

Supplementary Fig. 29 a Conductivity analysis of the ITO/SAM/Cu devices. **b** *J-V* curves of hole-only devices based on ITO/SAM/Cu. **c** SEM images of **c** Ph-4PACz and **d** Ph-Nd films deposited on ITO (scale bar: 100 nm). The dark *I-V* characteristics of hole-only devices display a clear Ohmic region at low bias voltage, indicating a satisfactory contact quality between the SAM and electrode, which is a prerequisite for applying the SCLC model.

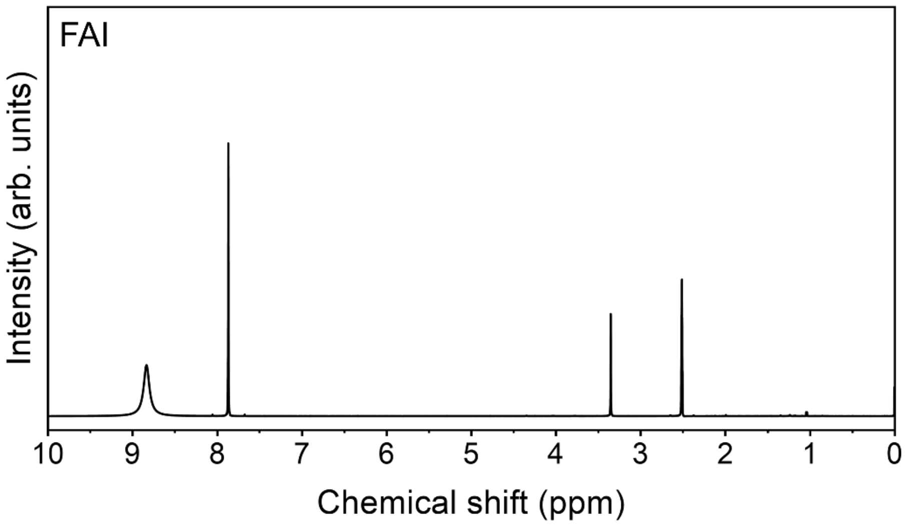

Supplementary Fig. 30 Liquid-state ^{1}H NMR spectrums of FAI (d-DMSO).

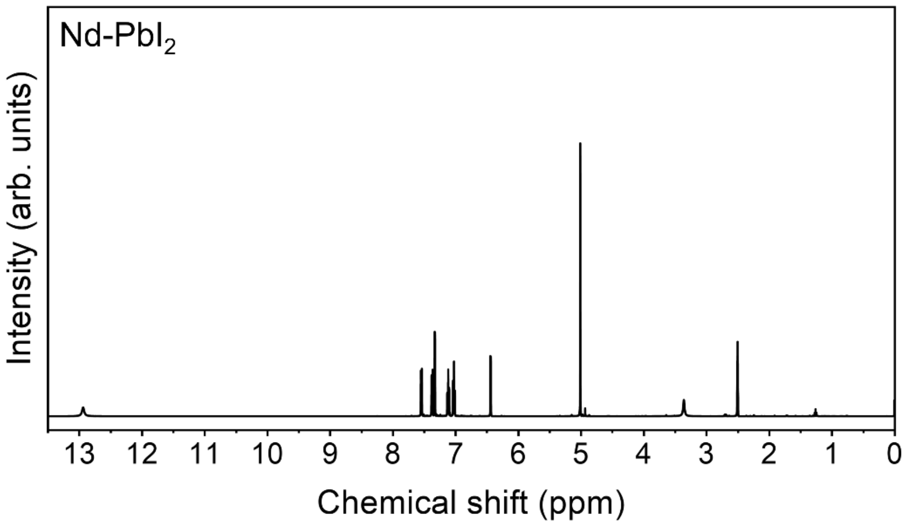

Supplementary Fig. 31 Liquid-state ^{1}H NMR spectrums of Nd-PbI_2 (d-DMSO).

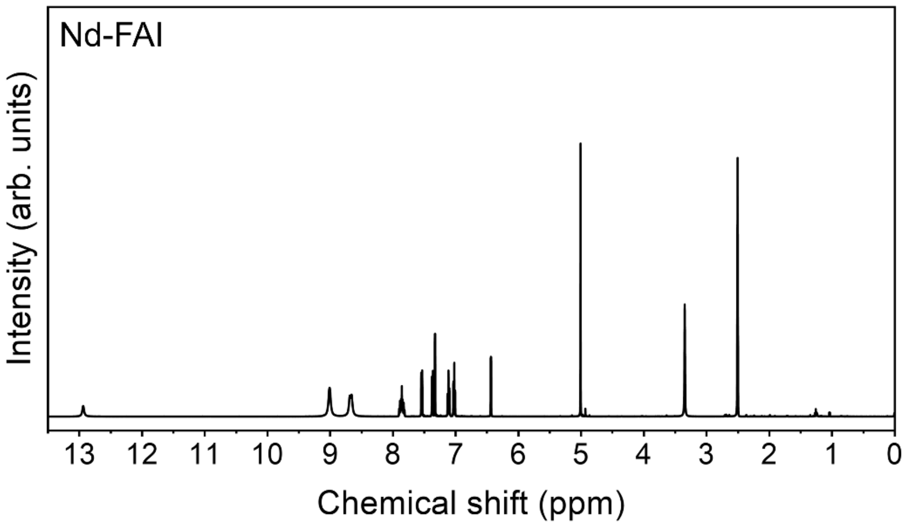

Supplementary Fig. 32 Liquid-state ^{1}H NMR spectrums of Nd-FAI (d-DMSO).

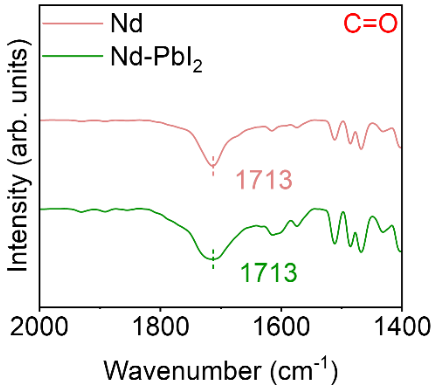

Supplementary Fig. 33 Partial enlarged FTIR spectra of Nd and Nd-PbI_2 powders.

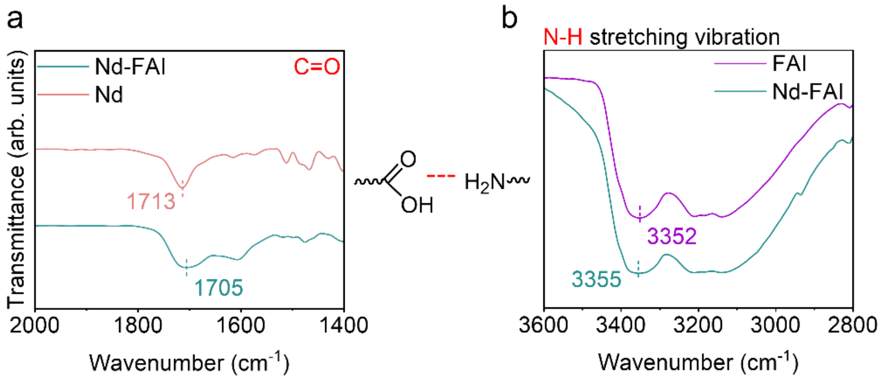

Supplementary Fig. 34 Partial enlarged FTIR spectra of Nd and Nd-FAI powders for **a** C=O stretching vibration and **b** N-H stretching vibration.

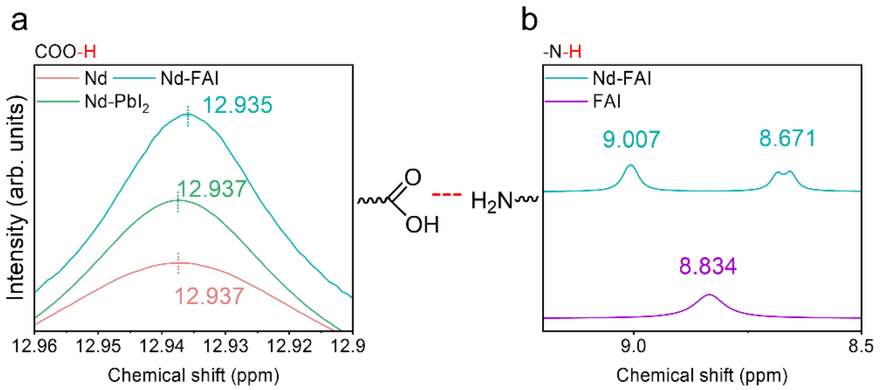

Supplementary Fig. 35 a Partial enlarged ^{1}H NMR spectra of Nd, Nd-PbI_2 and Nd-FAI solution. **b** Partial enlarged ^{1}H NMR spectra of Nd and Nd-FAI solution.

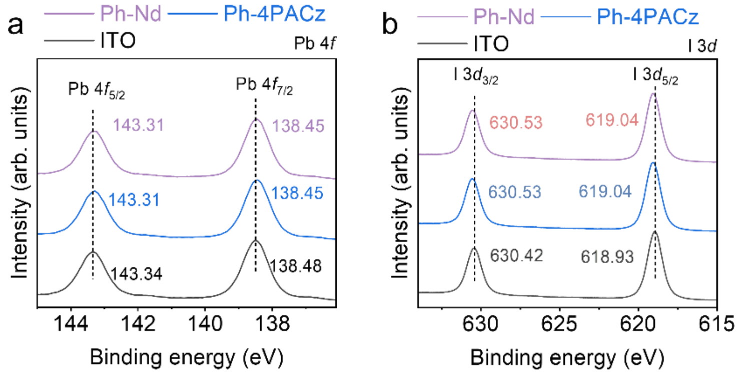

Supplementary Fig. 36 XPS spectra of **a** Pb 4*f* and **b** I 3*d* for the buried surface of perovskite films deposited on bare ITO, Ph-4PACz and Ph-Nd substrates.

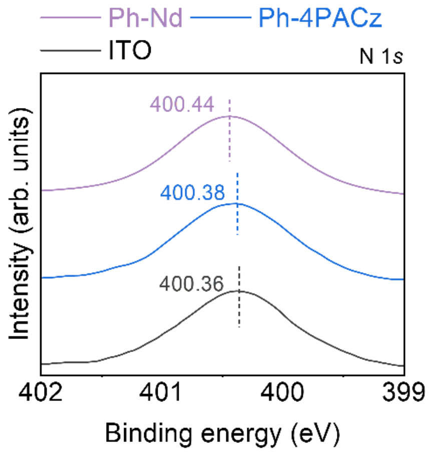

Supplementary Fig. 37 XPS spectra of N 1*s* for the buried surface of perovskite films deposited on bare ITO, Ph-4PACz and Ph-Nd substrates. Given that Nd is a weak acid (pKa ≈ 4.8), its carboxyl group exists predominantly as -COOH rather than -COO^{-}. The -COOH group has a much weaker coordinating ability toward lead cations compared to -COO^{-}, but instead readily forms hydrogen-bonding with FA cations.

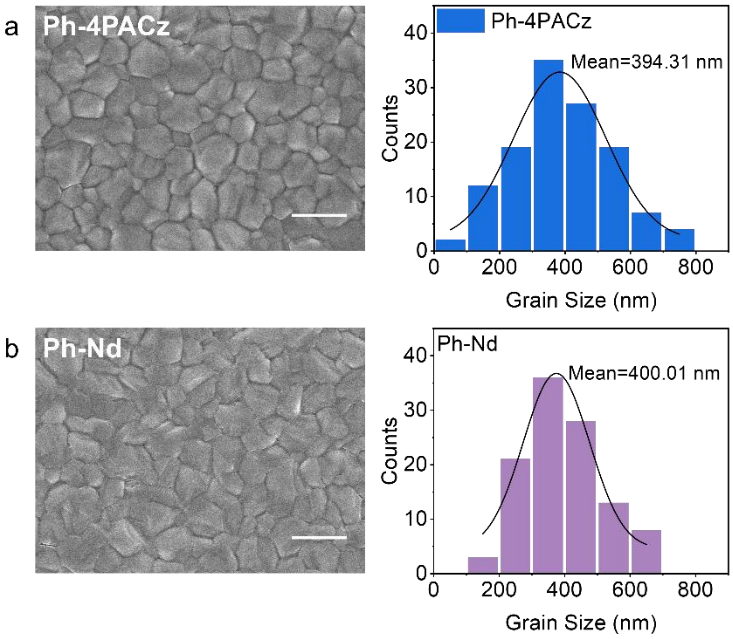

Supplementary Fig. 38 SEM images of the top surface of perovskite films on **a** Ph-4PACz and **b** Ph-Nd substrates (scale bar: 1μm) and the corresponding grain size distribution. The grain size of the perovskite film on Ph-Nd ranged from 176 to 652 nm, whereas a broader grain size range of 67-782 nm was observed for that on Ph-4PACz.

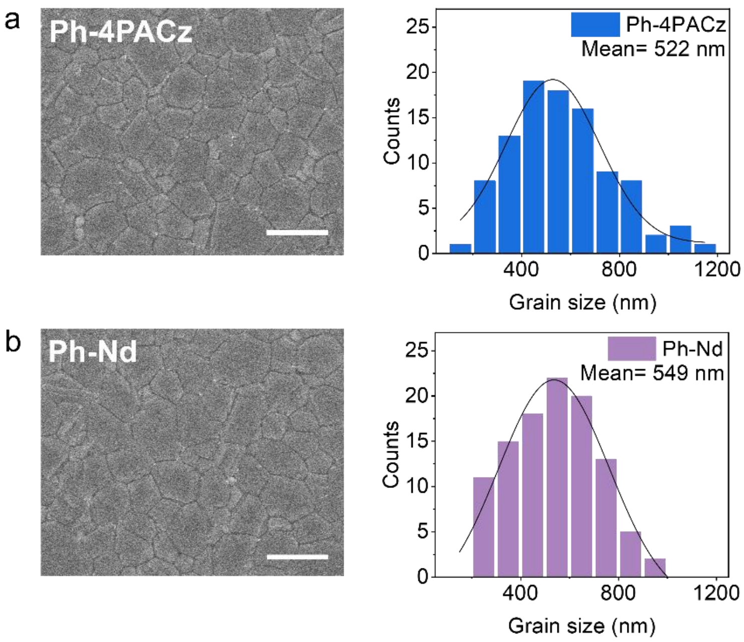

Supplementary Fig. 39 SEM images of the buried surface of perovskite films on **a** Ph-4PACz and **b** Ph-Nd substrates (scale bar: 1 μm) and the corresponding grain size distribution. The grain size of the perovskite film on Ph-4PACz ranged from 181 to 1112 nm, whereas a narrower grain size range of 207-997 nm was observed for that on Ph-Nd.

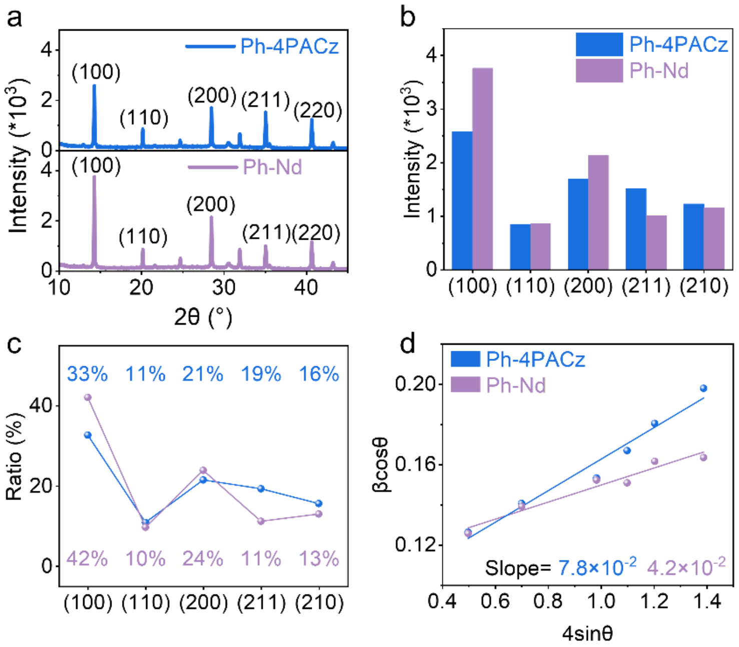

Supplementary Fig. 40 a XRD patterns of perovskite films on Ph-4PACz and Ph-Nd substrates. **b** Diffraction intensity of main diffraction peaks belonging to the (100), (110), (200), (211), (210) crystal planes and **c** the corresponding peak intensity ratio. Both (100) and (200) crystal planes belong to the {100} crystal plane family. **d** Williamson–Hall plots calculated from XRD patterns.

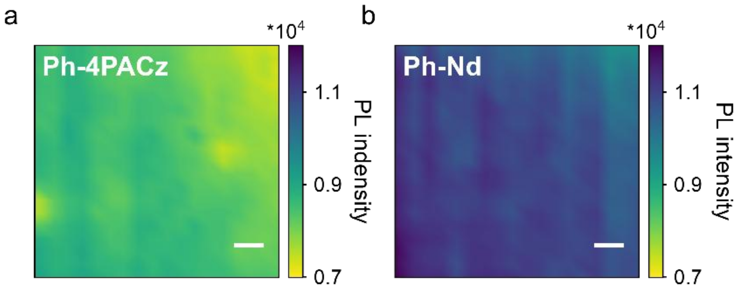

Supplementary Fig. 41 PL intensity mapping of perovskite films on **a** Ph-4PACz and **b** Ph-Nd substrates (scale bar: 1 μm).

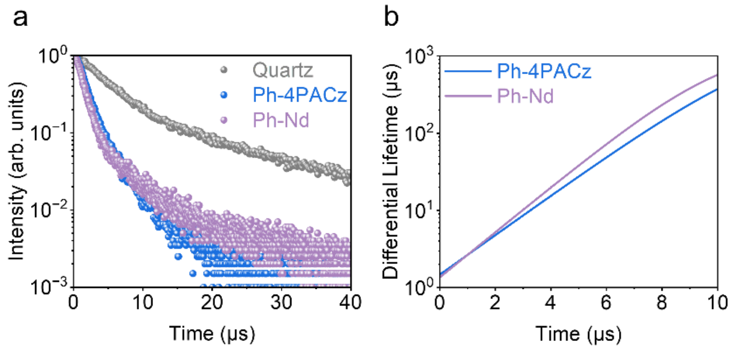

Supplementary Fig. 42 a TRPL spectra of perovskite films on bare quartz, Ph-4PACz, and Ph-Nd substrates. **b** First-order differential of TRPL spectra at the fast decay phase. The τ_1 corresponds to the fast decay phase, whereas the τ_2 belongs to the slow decay stage.

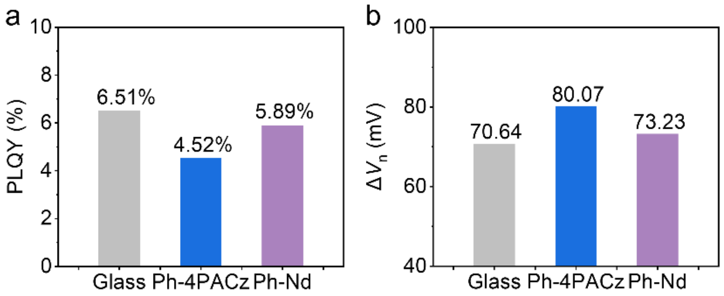

Supplementary Fig. 43 **a** PLQY values and **b** the corresponding calculated ΔV_n values of perovskite films on bare ITO, Ph-4PACz, and Ph-Nd substrates.

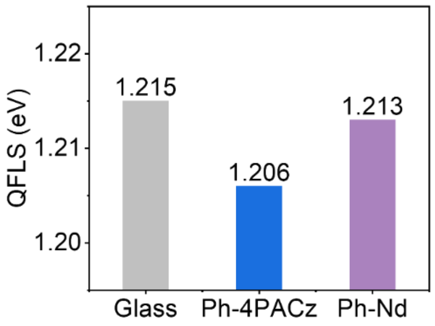

Supplementary Fig. 44 QFLS values of perovskite films on bare ITO, Ph-4PACz, and Ph-Nd substrates.

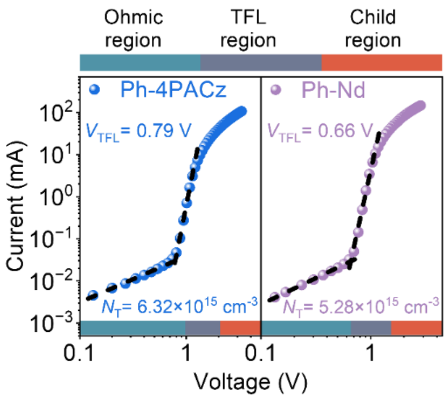

Supplementary Fig. 45 SCLC analysis of hole-only devices with a structure of ITO/SAM/perovskite/PTAA/Cu.

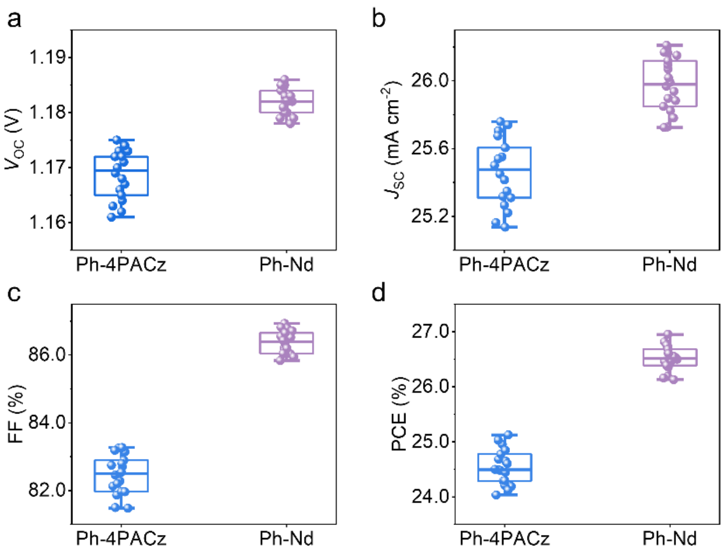

Supplementary Fig. 46 Statistics of photovoltaic parameters of Ph-4PACz and Ph-Nd devices (18 devices fabricated from the same batch).

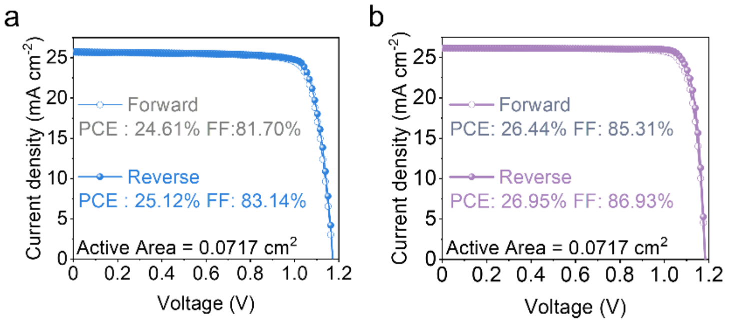

Supplementary Fig. 47 *J-V* curves of the device based on **a** Ph-4PACz and **b** Ph-Nd scanned under forward and reverse directions.

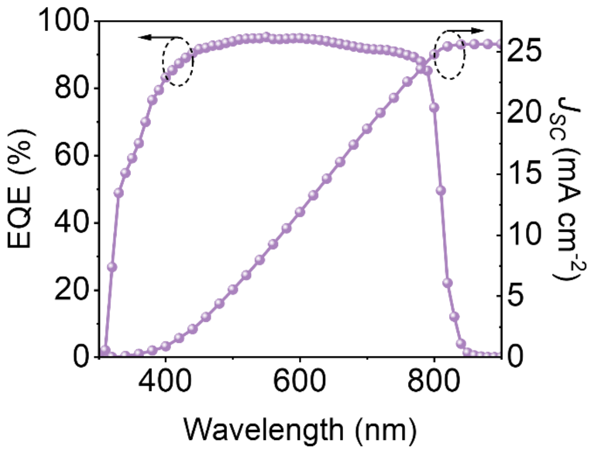

Supplementary Fig. 48 EQE spectra of the Ph-Nd device.

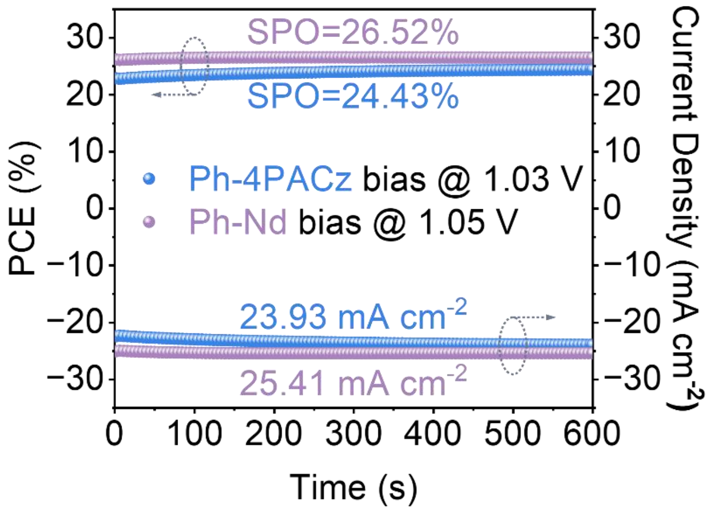

Supplementary Fig. 49 Stabilized power output at the MPPT.

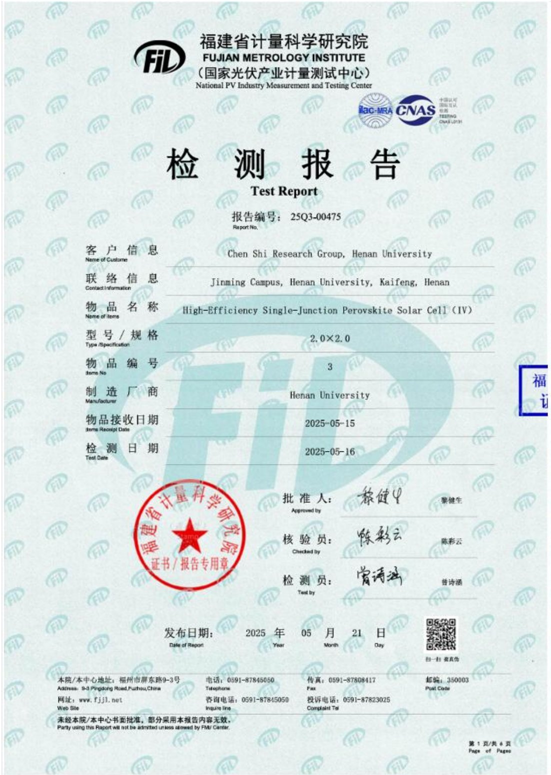

福建省计量科学研究院
FUJIAN METROLOGY INSTITUTE
（国家光伏产业计量测试中心）
National PV Industry Measurement and Testing Center

检 测 报 告
Test Report

报告编号：25Q3-00475
Report No.

客户信息 Name of Custome	Chen Shi Research Group, Henan University
联络信息 Contact Information	Jinming Campus, Henan University, Kaifeng, Henan
物品名称 Name of Items	High-Efficiency Single-Junction Perovskite Solar Cell (IV)
型号／规格 Type /Specification	2.0×2.0
物品编号 Items No	3
制造厂商 Manufacturer	Henan University
物品接收日期 Items Receipt Date	2025-05-15
检测日期 Test Date	2025-05-16

批准人：Approved by 黎健生
核验员：Checked by 陈彩云
检测员：Test by 曾诗涵

发布日期：Date of Report 2025 年 Year 05 月 Month 21 日 Day

扫一扫 查真伪

本院／本中心地址：福州市屏东路9-3号 Address: 9-3 Pingdong Road,Fuzhou,China
电话：0591-87845050 Telephone
传真：0591-87808417 Fax
邮编：350003 Post Code
网址：www.fjjl.net Web Site
咨询电话：0591-87845050 Inquire line
投诉电话：0591-87823025 Complaint Tel

未经本院／本中心书面批准，部分采用本报告内容无效。
Party using this Report will not be admitted unless allowed by FMI/ Center.

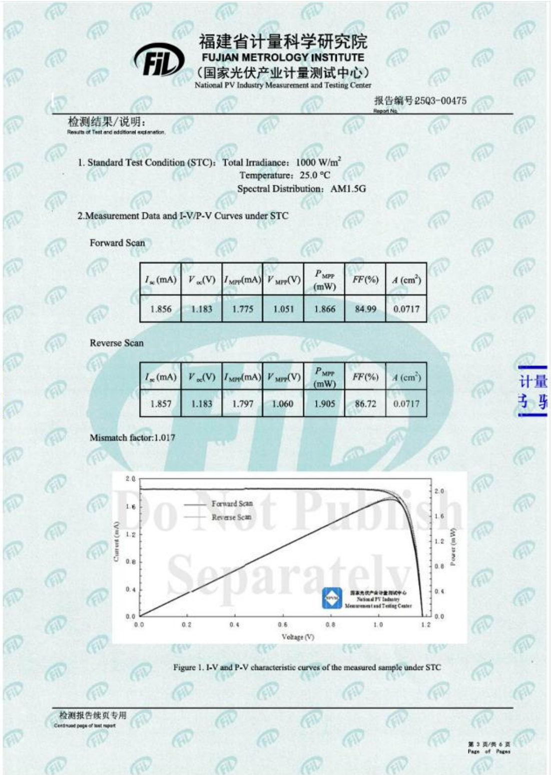

福建省计量科学研究院
FUJIAN METROLOGY INSTITUTE
（国家光伏产业计量测试中心）
National PV Industry Measurement and Testing Center

报告编号 25Q3-00475
Report No.

检测结果／说明：
Results of Test and additional explanation.

1. Standard Test Condition (STC): Total Irradiance: 1000 W/m²
Temperature: 25.0 °C
Spectral Distribution: AM1.5G

2.Measurement Data and I-V/P-V Curves under STC

Forward Scan

I_{sc} (mA)	V_{oc}(V)	I_{MPP}(mA)	V_{MPP}(V)	P_{MPP} (mW)	FF(%)	A (cm²)
1.856	1.183	1.775	1.051	1.866	84.99	0.0717

Reverse Scan

I_{sc} (mA)	V_{oc}(V)	I_{MPP}(mA)	V_{MPP}(V)	P_{MPP} (mW)	FF(%)	A (cm²)
1.857	1.183	1.797	1.060	1.905	86.72	0.0717

Mismatch factor:1.017

Figure 1. I-V and P-V characteristic curves of the measured sample under STC

检测报告续页专用
Continued page of test report

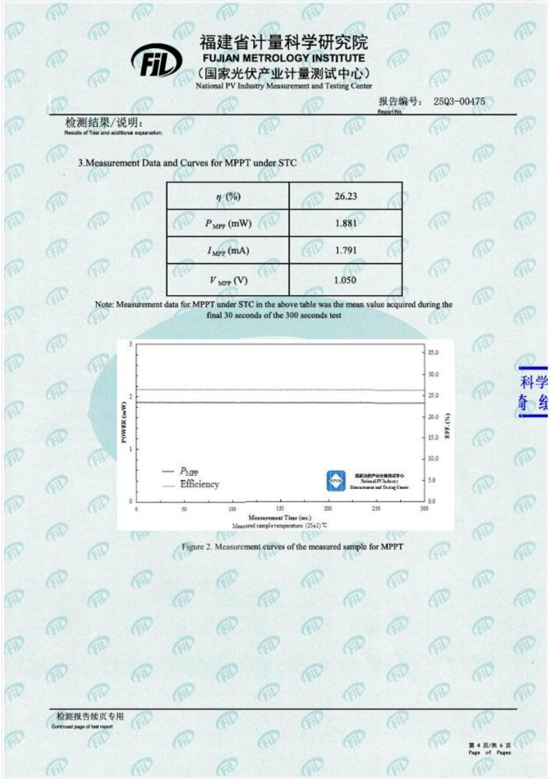

福建省计量科学研究院
FUJIAN METROLOGY INSTITUTE
（国家光伏产业计量测试中心）
National PV Industry Measurement and Testing Center

报告编号：25Q3-00475
Report No.

检测结果／说明：
Results of Test and additional explanation.

3.Measurement Data and Curves for MPPT under STC

η (%)	26.23
P_{MPP} (mW)	1.881
I_{MPP} (mA)	1.791
V_{MPP} (V)	1.050

Note: Measurement data for MPPT under STC in the above table was the mean value acquired during the final 30 seconds of the 300 seconds test

Figure 2. Measurement curves of the measured sample for MPPT

检测报告续页专用
Continued page of test report

Supplementary Fig. 50 The National PV Industry Measurement and Testing Center certification report of small area (0.0717 cm^2) PSCs based on Ph-Nd (Ph-4PACz:Nd 1:1). The certified *J-V* curves give a PCE of 26.57% (V_{OC} = 1.183 V, J_{SC} = 25.90 mA cm^{-2}, FF = 86.72%).

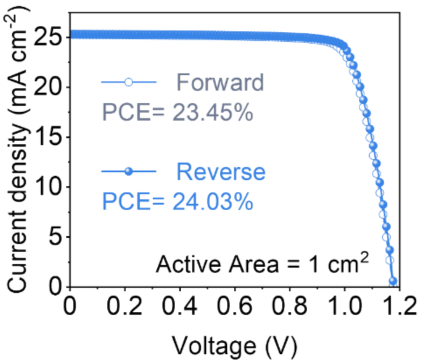

Supplementary Fig. 51 *J*-*V* curves of 1 cm^2 Ph-4PACz PSCs under forward and reverse scan.

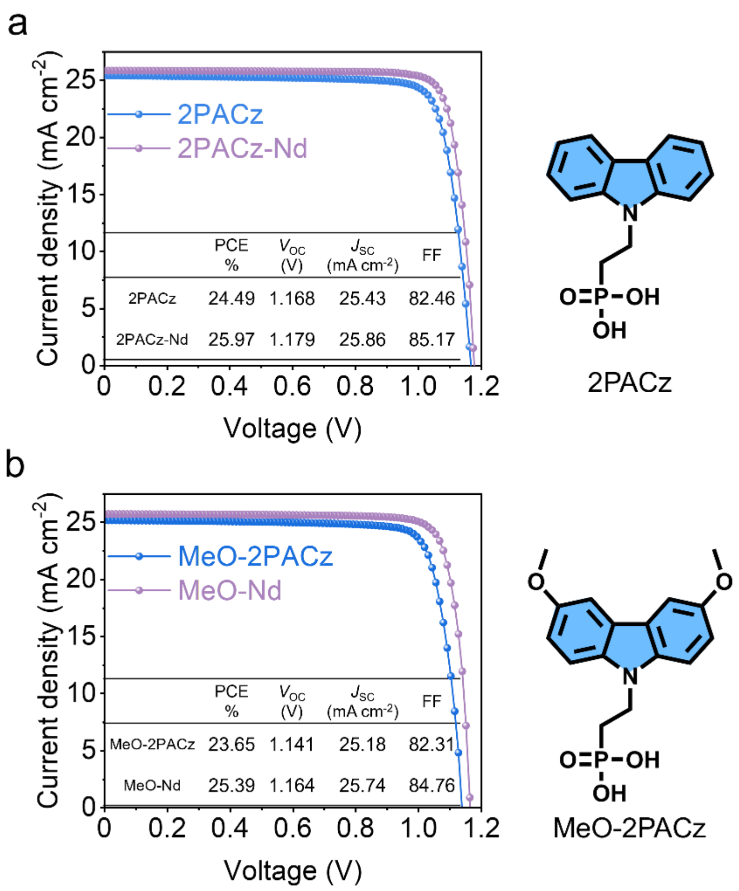

Supplementary Fig. 52 a *J*-*V* curves of 2PACz based devices without and with Nd insertion. **b** *J*-*V* curves of MeO-2PACz based devices without and with Nd insertion.

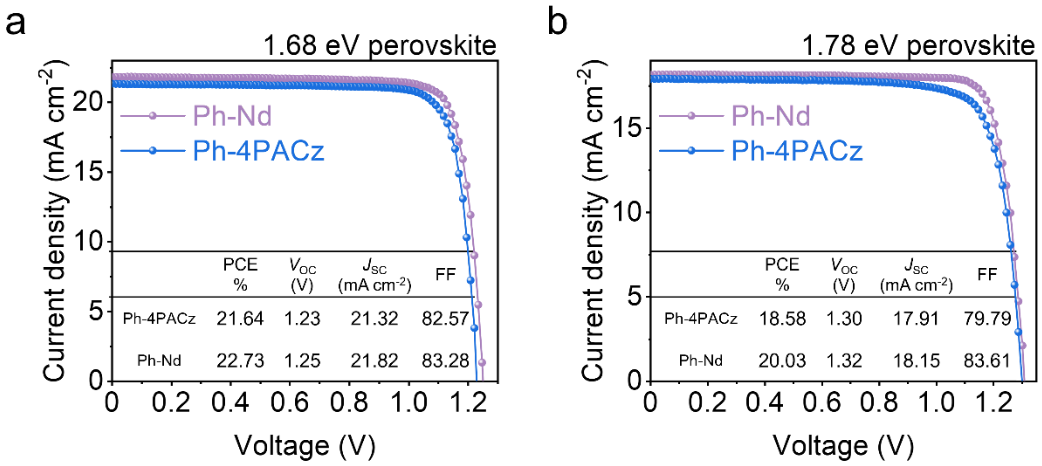

Supplementary Fig. 53 a *J*-*V* curves of 1.68 eV band-gap PSCs based on Ph-4PACz and Ph-Nd. **b** *J*-*V* curves of 1.78 eV band-gap PSCs based on Ph-4PACz and Ph-Nd.

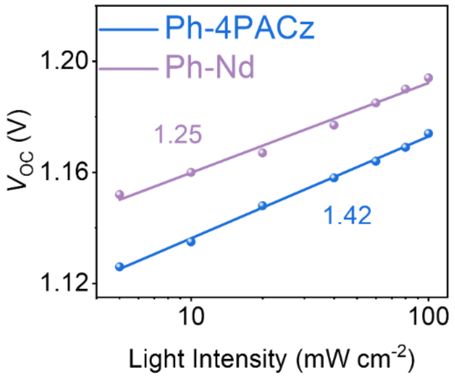

Supplementary Fig. 54 Light-intensity-dependent V_{OC} of Ph-4PACz and Ph-Nd devices.

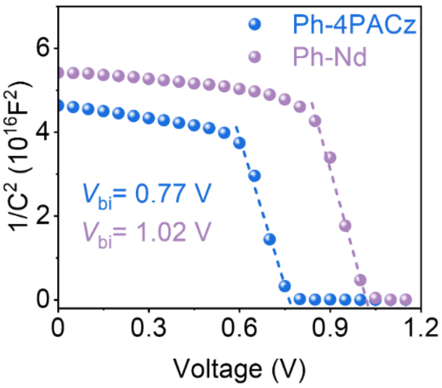

Supplementary Fig. 55 Mott-Schottky plots of Ph-4PACz and Ph-Nd devices.

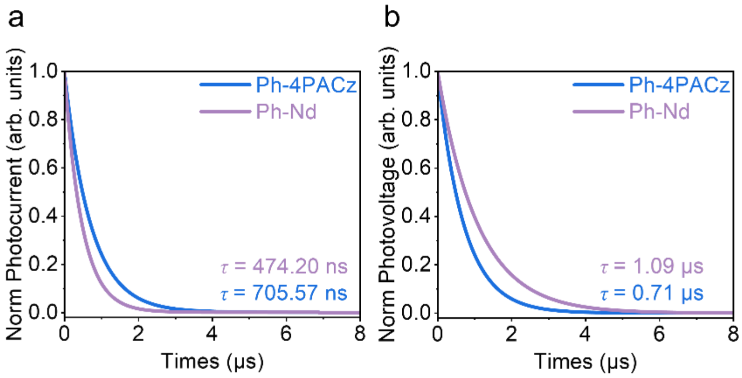

Supplementary Fig. 56 a TPC and **b** TPV curves of Ph-4PACz and Ph-Nd devices.

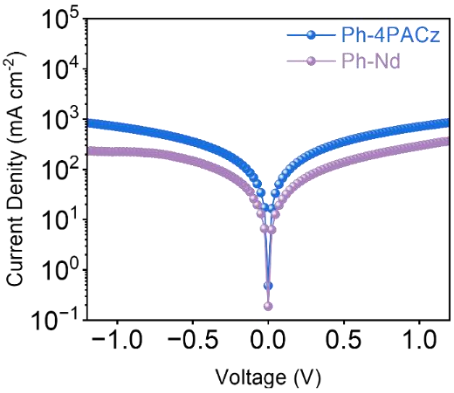

Supplementary Fig. 57 Dark *J-V* curves of Ph-4PACz and Ph-Nd devices.

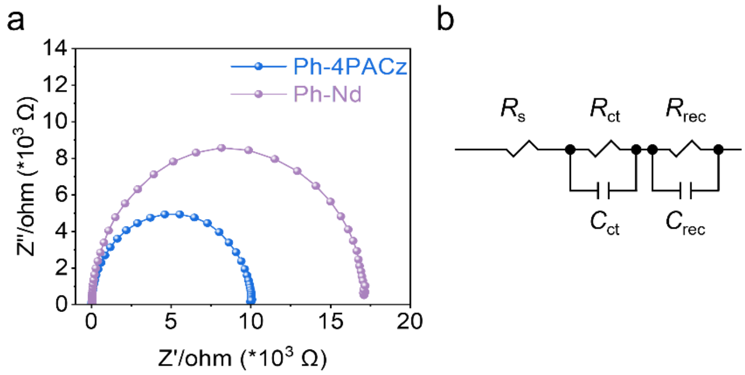

Supplementary Fig. 58 a Nyquist plots of Ph-4PACz and Ph-Nd devices and **b** Schematic illustration of an equivalent circuit model. R_{ct} and R_{rec} are fitted form high-frequency and low-frequency regions, respectively.

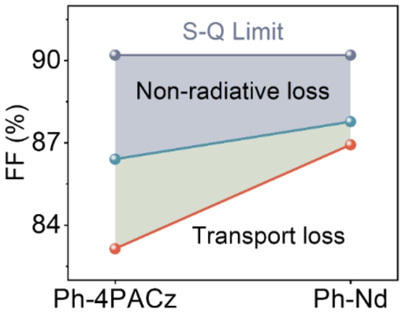

Supplementary Fig. 59 FF loss analysis of Ph-4PACz and Ph-Nd devices.

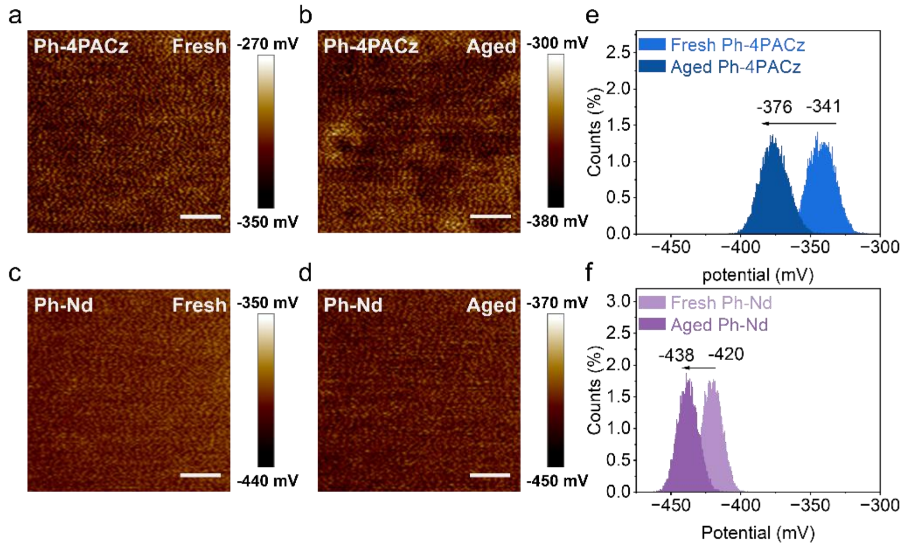

Supplementary Fig. 60 KPFM images of Ph-4PACz (**a-b**) and Ph-Nd films (**c-d**) (scale bar: 1 μm). Contact potential difference distributions of Ph-4PACz (**e**) and Ph-Nd (**f**) films before and after aging extracted from KPFM results. To directly compare the intrinsic thermal stability of SAM layers, we implemented Kelvin probe force microscopy measurements for Ph-4PACz and Ph-Nd films before and after thermal aging (65 °C for 28 days in N_2 atmosphere). This thermal aging led a reduction of 35 mV in contact potential difference for the Ph-4PACz film, much larger than that (18 mV) of the Ph-Nd film, suggesting better stability of the alternating co-adsorbed SAM.

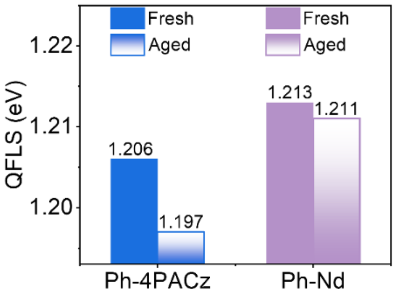

Supplementary Fig. 61 QFLS values of perovskite films on fresh or aged Ph-4PACz and Ph-Nd substrates. The perovskite deposited on the aged (65 °C for 28 days in N_2 atmosphere) Ph-Nd film achieved a high QFLS value of 1.211 eV, comparable to that (1.213 eV) on the fresh substrate, manifesting stable interfacial contact between perovskite and Ph-Nd. In contrast, the QFLS of the perovskite grown on the fresh Ph-4PACz film significantly decreased from 1.206 to 1.197 eV when replacing with the aged one, suggesting SAM-aging-induced enhanced interfacial non-radiative recombination. These findings collectively provide direct experimental evidence that the improved stability of the Ph-Nd device is high associated with the enhanced thermal and electronic stability of the alternating co-adsorbed SAM.

Supplementary Tables

Supplementary Table 1 Summary of contact Angle and interaction parameter χ.

SAM	H_2O(°)	EG(°)	system	χ
Ph-4PACz	73.6	55.8	Ph-4PACz/Nd	0.30
Nd	68.2	48.7	2PACz/N-Id	0.20
2PACz	74.3	56.6	Ph-4P/2PACz	0.16

Supplementary Table 2 HOMO levels of Ph-Nd in different mixing ratios and perovskite.

Film	$HOMO^{CV}$ (eV)	$HOMO^{UPS}$ (eV)	Fermi level (eV)
Ph-Nd 1:0	-5.25	-5.51	-4.87
1:0.5	-5.27	-5.61	-5.00
1:1	-5.29	-5.65	-5.01
1:2	-5.26	-5.62	-5.00
0:1	-5.17	-5.35	-4.39
perovskite	/	-5.67	/
ITO	/	-7.61	/

Supplementary Table 3 Photovoltaic parameters of devices based on SAMs with different mixing ratios.

Sample (Ph-Nd)	V_{OC} (V)	J_{SC} (mA cm^{-2})	FF (%)	PCE (%)
1:0	1.168±0.007	25.45±0.31	82.38±0.89	24.57±0.55
1:0.5	1.173±0.006	25.75±0.27	85.23±0.73	25.79±0.48
1:1	1.182±0.004	25.96±0.25	86.38±0.55	26.54±0.41
1:2	1.168±0.008	25.58±0.30	85.62±0.68	25.65±0.52
0:1	1.005±0.019	24.05±0.36	80.73±1.79	19.54±1.09

Supplementary Table 4 Parameters of SAM films obtained from GIWAXS data.

Film	q-location ($Å^{-1}$)	d-spacing[a] (Å)
Ph-4PACz	-	-
Ph-Nd	0.69	9.1
	1.38	-

[a] Stacking distance is calculated using the formula $d=2\pi/q$

Supplementary Table 5 XRD intensity and full width at half maxima (FWHM) extracted from XRD results.

	Ph-4PACz/perovskite			Ph-Nd/perovskite		
	FWHM	Intensity	Ratio (%)	FWHM	Intensity	Ratio (%)
(100)	0.12729	2580	32.7	0.12662	3761	42.2
(110)	0.14285	857	10.9	0.14130	868	9.7
(200)	0.15501	1696	21.4	0.152592	2139	23.9
(211)	0.17308	1525	19.3	0.15565	1006	11.2
(210)	0.18979	1232	15.7	0.20632	1160	13.0

Supplementary Table 6 Summary of fitting parameters based on TRPL spectra.

Substrate	A_1	τ_1 (μs)	A_2	τ_2 (μs)	τ_{avg} (μs)	SRV (m/s)
Ph-4PACz	1.15	1.69	0.02	12.68	3.18	0.15
Ph-Nd	1.09	1.48	0.03	17.76	5.31	0.07

Supplementary Table 7 Photovoltaic parameters of Ph-4PACz and Ph-Nd based devices.

Device	V_{OC} (V)	J_{SC} (mA cm^{-2})	FF (%)	PCE (%)
Ph-4PACz	1.168±0.007	25.45±0.31	82.38±0.89	24.57±0.55
champion	1.174	25.74	83.14	25.12
Ph-Nd	1.182±0.004	25.96±0.25	86.38±0.55	26.54±0.41
champion	1.185	26.16	86.93	26.95

Supplementary Table 8 Photovoltaic parameters of the champion devices (0.0717 cm^2) based on Ph-4PACz and Ph-Nd under different scan directions.

Device	Scan direction	V_{OC} (V)	J_{SC} (mA cm^{-2})	FF (%)	PCE (%)	Hysteresis (%)
Ph-4PACz	Forword	1.173	25.68	81.70	24.61	2.04
	Reverse	1.174	25.74	83.14	25.12	
Ph-Nd	Forword	1.184	26.18	85.31	26.44	1.87
	Reverse	1.185	26.16	86.93	26.95	

Supplementary Table 9 Photovoltaic parameters of the large-area (1 cm^2) devices based on Ph-Nd under different scan directions.

Device	Scan direction	V_{OC} (V)	J_{SC} (mA cm^{-2})	FF (%)	PCE (%)	Hysteresis (%)
Ph-4PACz	Forword	1.176	25.26	78.93	23.45	2.41
	Reverse	1.178	25.32	80.55	24.03	
Ph-Nd	Forword	1.186	25.98	81.79	25.20	1.59
	Reverse	1.187	26.04	82.85	25.61	

Supplementary Table 10 EIS fitting parameters of devices based on Ph-4PACz and Ph-Nd.

Substrate	R_S (Ω)	R_{ct} (Ω)	R_{rec} (Ω)
Ph-4PACz	31.4	383.5	9513
Ph-Nd	21.6	115.4	16877

Supplementary References

1. Sumita, M. et al. Dispersion of fillers and the electrical conductivity of polymer blends filled with carbon black. *Polym. Bull.* **25**, 265-271, (1991).
2. Park, S. M. et al. Low-loss contacts on textured substrates for inverted perovskite solar cells. *Nature* **624**, 289-294, (2023).
3. Liu, M. et al. Compact Hole-Selective Self-Assembled Monolayers Enabled by Disassembling Micelles in Solution for Efficient Perovskite Solar Cells. *Adv. Mater.* **35**, 2304415, (2023).
4. Zheng, Z. et al. Pre-Buried Additive for Cross-Layer Modification in Flexible Perovskite Solar Cells with Efficiency Exceeding 22%. *Adv. Mater.* **34**, 2109879, (2022).
5. Liu, S. et al. Buried interface molecular hybrid for inverted perovskite solar cells. *Nature* **632**, 536-542, (2024).
6. Kong, W. et al. Organic Monomolecular Layers Enable Energy-Level Matching for Efficient Hole Transporting Layer Free Inverted Perovskite Solar Cells. *ACS Nano* **13**, 1625-1634, (2019).
7. Li, C. et al. Fully Aromatic Self-Assembled Hole-Selective Layer toward Efficient Inverted Wide-Bandgap Perovskite Solar Cells with Ultraviolet Resistance. *Angew. Chem., Int. Ed.* **63**, e202315281, (2024).
8. Zhang, Z. et al. Anchoring Charge Selective Self-Assembled Monolayers for Tin–Lead Perovskite Solar Cells. *Adv. Mater.* **36**, 2312264, (2024).
9. Zhu, P. et al. Aqueous synthesis of perovskite precursors for highly efficient perovskite solar cells. *Science* **383**, 524-531, (2024).
10. Geng, S. et al. Revealing Collaborative Effects of Binary Additives on Regulating Precursor Crystallization Toward Highly Efficient Perovskite Solar Cells. *Angew. Chem., Int. Ed.* **64**, e202424910, (2025).
11. Li, Z. et al. Stabilized hole-selective layer for high-performance inverted p-i-n perovskite solar cells. *Science* **382**, 284-289, (2023).
12. Lu, T. et al. Independent gradient model based on Hirshfeld partition: A new method for visual study of interactions in chemical systems. *J. Comput. Chem.* **43**, 539-555,

(2022).
