# Supplementary material for: Homogenizing interfacial assembly via indole-mediated binary monolayers for perovskite solar cells
Source: Nat Commun. 2026 Apr 2;17:4742. doi: 10.1038/s41467-026-71301-6 (PMC13216523; doi:10.1038/s41467-026-71301-6)
Supplement: Supplementary file 2 — Reporting Summary [file 41467_2026_71301_MOESM2_ESM.pdf]

nature portfolio

Corresponding author(s): Nan Shen; Xingzhu Wang; Geping Qu; Shi Chen; Mohammad Khaja Nazeeruddin

Solar Cells Reporting Summary

Nature Portfolio wishes to improve the reproducibility of the work that we publish. This form is intended for publication with all accepted papers reporting the characterization of photovoltaic devices and provides structure for consistency and transparency in reporting. Some list items might not apply to an individual manuscript, but all fields must be completed for clarity.

For further information on Nature Research policies, including our data availability policy, see Authors & Referees.

Experimental design

Please check the following details are reported in the manuscript, and provide a brief description or explanation where applicable.

1. Dimensions

Area of the tested solar cells

- [x] Yes — 0.0717 cm2 for small-area device and 1cm2 for large-area device.
- [ ] No — *Explain why this information is not reported/not relevant.*

Method used to determine the device area

- [x] Yes — A metal mask with an area of 0.0717cm2 and 1cm2 was used to define the active area of small cell.
- [ ] No — *Explain why this information is not reported/not relevant.*

2. Current-voltage characterization

Current density-voltage (J-V) plots in both forward and backward direction

- [x] Yes — The manuscript provides current density - voltage (J-V) plots in both forward and backward direction (Fig. 4b and Supplementary Fig. 47).
- [ ] No

Voltage scan conditions

- [x] Yes — J-V curves were measured by both forward (from 0 to 1.2 V) and reverse scanning (from 1.2 to 0 V) in a scanning rate of 100 mV/s.
- [ ] No — *Explain why this information is not reported/not relevant.*

Test environment

- [x] Yes — The current-voltage characteristics were obtained in glove box at room temperature.
- [ ] No — *Explain why this information is not reported/not relevant.*

Protocol for preconditioning of the device before its characterization

- [ ] Yes — *Provide a description of the protocol.*
- [x] No — No specific protocol for preconditioning was applied.

Stability of the J-V characteristic

- [x] Yes — Maximum power point tracking and heat stability tests were conducted, as reported in the manuscript (Figs. 4f-g).
- [ ] No — *Explain why this information is not reported/not relevant.*

3. Hysteresis or any other unusual behaviour

Description of the unusual behaviour observed during the characterization

- [x] Yes — Low hysteresis was observed upon altering the scan direction, as reported in the manuscript.
- [ ] No — *Explain why this information is not reported/not relevant.*

Related experimental data

- [x] Yes — Supplementary Fig. 47 and Supplementary Table 8.
- [ ] No — *Explain why this information is not reported/not relevant.*

4. Efficiency

External quantum efficiency (EQE) or incident photons to current efficiency (IPCE)

- [x] Yes — EQE spectra were recorded, as reported in Supplementary Fig. 48.
- [ ] No — *Explain why this information is not reported/not relevant.*

A comparison between the integrated response under the standard reference spectrum and the response measure under the simulator

- [x] Yes — The integrated JSC from EQE is consistent with those from J-V testing (within 3% error).
- [ ] No — *Explain why this information is not reported/not relevant.*

For tandem solar cells, the bias illumination and bias voltage used for each subcell	☐ Yes<br>☒ No	*Provide a description of the measurement conditions.*<br>Not applicable as no tandem solar cells are reported in this work.

5. Calibration

Light source and reference cell or sensor used for the characterization	☒ Yes<br>☐ No	The current density–voltage (J-V) characteristics of the devices were measured in glove box by an AM 1.5G solar simulator and a Keithley 2400 source meter.<br>*Explain why this information is not reported/not relevant.*
Confirmation that the reference cell was calibrated and certified	☒ Yes<br>☐ No	The standard Si reference cell was purchased from Enli Tech. Co., Ltd., Taiwan, which was calibrated and certified by NREL.<br>*Explain why this information is not reported/not relevant.*
Calculation of spectral mismatch between the reference cell and the devices under test	☒ Yes<br>☐ No	The spectral mismatch between our simulator and the AM 1.5 solar source was insignificant as the integrated current densities estimated from the EQE spectra were in good agreement with the values obtained from the current density - voltage (J-V) curves as detailed in the manuscript.<br>*Explain why this information is not reported/not relevant.*

6. Mask/aperture

Size of the mask/aperture used during testing	☒ Yes<br>☐ No	0.0717 cm2 for small-area device and 1cm2 for large-area device.<br>*Explain why this information is not reported/not relevant.*
Variation of the measured short-circuit current density with the mask/aperture area	☐ Yes<br>☒ No	*Report the difference in the short-circuit current density values measured with the mask and aperture area.*<br>The mask/aperture area is fixed and cannot change.

7. Performance certification

Identity of the independent certification laboratory that confirmed the photovoltaic performance	☒ Yes<br>☐ No	The photovoltaic performance of our devices were certified by National PV Industry Measure and Testing Center, Fujian Metrology Institute.<br>*Explain why this information is not reported/not relevant.*
A copy of any certificate(s)	☒ Yes<br>☐ No	Supplementary Fig. 50.<br>*Explain why this information is not reported/not relevant.*

8. Statistics

Number of solar cells tested	☒ Yes<br>☐ No	18 devices for each condition were tested, as reported (Supplementary Fig. 46 and Supplementary Table 7).<br>*Explain why this information is not reported/not relevant.*
Statistical analysis of the device performance	☒ Yes<br>☐ No	Supplementary Fig. 46 and Supplementary Table 7.<br>*Explain why this information is not reported/not relevant.*

9. Long-term stability analysis

Type of analysis, bias conditions and environmental conditions	☒ Yes<br>☐ No	The long-term stability under N2 and 1 sun-equivalent LED illumination for unencapsulated devices (reaching an operating temperature of ~60 ±5°C). Thermal stability assessments under N2 and 85 °C for unencapsulated devices.<br>*Explain why this information is not reported/not relevant.*
